# Ethnomedicinal Uses, Phytochemistry and Bioactivities of Halophytes and Salt-Tolerant Plants with Potential Against Metabolic Syndrome: A Comprehensive Review

**DOI:** 10.3390/md24080262

**Published:** 2026-07-29

**Authors:** Maria João Rodrigues, Pedro García-Caparrós, Catarina Guerreiro Pereira, Christian Magné, Karim Ben Hamed, Mariana Laundry de Mesquita, Luísa Custódio

**Affiliations:** 1 Centre of Marine Sciences (CCMAR/CIMAR LA), Faculty of Sciences and Technology, University of Algarve, Campus of Gambelas, Building 7, 8005-139 Faro, Portugal; mjrodrigues@ualg.pt (M.J.R.); cagpereira@ualg.pt (C.G.P.); 2 Higher Polytechnic School of Engineering, University of Almería, Carretera Sacramento s/n, La Cañada de San Urbano, 04120 Almeria, Spain; pgc993@ual.es; 3 Géoarchitecture Laboratory—Territories, Urban Planning, Biodiversity and Environment, UFR Sciences et Techniques, Université de Bretagne Occidentale, 6 Avenue Victor Le Gorgeu, CS 93897, 29238 Brest Cedex 3, France; christian.magne@univ-brest.fr; 4 National Engineering School of Manouba (ENIM), University of Manouba, Campus Universitaire de la Manouba, Manouba 2010, Tunisia; karim.benhamed@mse.uma.tn; 5 Laboratory of Extremophile Plants, Centre of Biotechnology of Borj Cedria, BP 95, Hammam-Lif 2050, Tunisia; 6 Department of Pharmacy, Faculty of Health Sciences, University of Brasília, Campus Universitário Darcy Ribeiro, Brasília 70910-900, Brazil; marilaundry@gmail.com

**Keywords:** diabetes, halophytes, inflammation, obesity, cardiovascular diseases, secondary metabolites

## Abstract

Metabolic syndrome (MetS) is a complex cluster of interconnected metabolic abnormalities, including central obesity, insulin resistance, dyslipidemia, hypertension, chronic inflammation, and impaired glucose metabolism, which collectively increase the risk of type 2 diabetes and cardiovascular diseases. Although current therapeutic strategies, including lifestyle interventions and pharmacological treatments, can be effective in managing specific MetS components, the multifactorial nature of this condition continues to encourage the search for complementary approaches and novel bioactive compounds. Salt-tolerant plants (STPs), particularly halophytes, survive under adverse saline and oxidative stress conditions through specialized physiological and biochemical adaptations, including the production of diverse secondary metabolites such as phenolic acids, flavonoids, sterols, terpenoids and polysaccharides. Several of these compounds have been associated with health-promoting properties, including antioxidant, anti-inflammatory, antidiabetic, antihypertensive, lipid-modulating and cardioprotective effects. This review provides an integrated and critical overview of the ethnomedicinal uses, phytochemistry and bioactivities of STPs with potential relevance to MetS and its associated conditions, namely chronic inflammation, diabetes, hypertension, cardiovascular disorders, dyslipidemia and obesity. The review first introduces the main features of MetS and the relevance of STPs as bioresources, followed by a synthesis of ethnomedicinal uses related to MetS-associated disorders. It then discusses *in vitro* and *in vivo* evidence for selected species, extracts and compounds, including reported bioactive metabolites and proposed mechanisms of action when available. Finally, the most promising species, extracts and metabolites are highlighted, together with current limitations and future research needs regarding efficacy, safety, bioavailability, standardization and clinical relevance.

## 1. Introduction

Metabolic syndrome (MetS) is recognized as one of the most significant public health challenges in both developed and developing nations, affecting circa 30% of the global population [1]. It is a complex cluster of metabolic abnormalities, including central obesity, insulin resistance, dyslipidemia, hypertension, and chronic inflammation, which collectively elevate the risk of cardiovascular diseases and type 2 diabetes (T2DM) [2,3]. Natural products have gained attention for their potential in managing MetS, owing to their diverse bioactive compounds capable of modulating metabolic pathways. Plant-derived substances, such as polyphenols and flavonoids, have demonstrated efficacy in improving insulin sensitivity, reducing oxidative stress, and attenuating inflammation, thereby addressing key components of MetS [4]. This therapeutic approach aligns with the increasing preference for natural and holistic health interventions.

Salt-tolerant plants (STPs) include a broad range of species capable of withstanding saline conditions. Among them, halophytes represent the most specialized and ecologically relevant group for bioprospecting in marine and marine-influenced environments. Halophytes are commonly defined as plants able to grow and complete their life cycle under high salinity, often at NaCl concentrations around or above 200 mM [5]. In the context of this review, the term STPs is therefore used to encompass halophytes and closely related salt-adapted species, with particular emphasis on plants naturally occurring in coastal and saline ecosystems shaped by marine influence. These include coastal salt marshes, estuaries, tidal flats, dunes, saline wetlands, mangrove-associated areas, and coastal or inland salt-affected soils influenced by seawater intrusion, salt spray, tidal flooding, or high evaporation rates. As such, STPs constitute a distinctive interface between terrestrial and marine ecosystems, being continuously exposed to salinity, waterlogging, oxidative stress, nutrient fluctuations, and other environmental pressures typical of marine-influenced habitats.

To survive and reproduce under these challenging conditions, STPs have evolved specialized physiological and biochemical mechanisms, including ion compartmentalization, osmotic adjustment, antioxidant defense, salt gland or bladder-mediated ion regulation, and the accumulation of protective secondary metabolites. These adaptations are closely associated with the biosynthesis of structurally diverse bioactive compounds, including phenolic acids, flavonoids, alkaloids, terpenoids, sterols, polysaccharides, and other specialized metabolites, many of which have been linked to antioxidant, anti-inflammatory, antidiabetic, antihypertensive, cardioprotective, and anti-obesity effects [6,7]. Accordingly, STPs represent a promising yet still underexplored reservoir of natural products with potential value for the development of novel therapeutic, nutraceutical, and health-promoting agents targeting MetS-related disorders.

Although several review papers have addressed the general biological properties and chemical constituents of STPs [6,8,9,10,11,12,13,14,15,16], none have specifically focused on their potential role in targeting MetS. This review aims to fill this gap by providing a comprehensive overview of the potential application of natural products from STPs in the management of key health disorders associated with MetS, including chronic inflammation, diabetes, hypertension, cardiovascular diseases, and obesity, while highlighting their relevance as marine-influenced bioresources for sustainable bioprospecting. It should be noted that this review does not aim to assess the nutritional suitability of STPs as whole foods for MetS management. Instead, it focuses on their ethnomedicinal relevance, phytochemical composition and bioactivities as potential sources of natural products, extracts, isolated compounds or standardized bioactive preparations with relevance to MetS-associated pathways.

### 1.1. An Overview of MetS

The term MetS, also known as ‘syndrome X’, was first introduced in 1988 as a cluster of related abnormalities, including hyperglycemia, dyslipidemia, and high blood pressure that could lead to an increased risk of cardiovascular diseases (CVDs) and that are possibly caused by underlying insulin resistance [2,3]. The definitive importance of MetS is due to its ability to identify individuals who are at high risk of T2DM and CVDs [2,3]. A meeting between the IDF Task Force on Epidemiology and Prevention, the National Heart, Lung, and Blood Institute (NHLBI), the American Heart Association (AHA), the World Heart Federation (WHF), and the International Atherosclerosis Society (IAS) resulted in a consensus stating that there should not be a mandatory diagnostic criterion and that waist circumference (central obesity) should be the main screening tool [17]. MetS is therefore defined by a group of parameters, including high waist circumference (male: 102 cm, female: 88 cm), high triglyceride (TG: 150 mg/dL), or take medications to lower TG, low high density lipoprotein cholesterol (-C) (male < 40 mg/dL, female < 50 mg/dL), high blood pressure (systolic blood pressure: 130 mm Hg, diastolic blood pressure: 85 mm Hg), or take medications to lower blood pressure and high fasting blood glucose (100 mg/dL), or take medications for diabetes [18,19].

MetS is a major public health concern worldwide, as it affects circa 30% of the global population [1,20]. It is considered a result of societal modernization, which is characterized by a rise in sedentarism, and its etiology includes an interconnection between metabolic, genetic, and environmental factors [2,3]. There are several mechanisms involved in MetS pathogenesis, whose contribution is still unclear [21], and the pathophysiology and pathogenesis of MetS have been reviewed by different authors, such as Adetunji et al. [22], Fahed et al. [2], and Marzoog [23]. The underlying basis of MetS are increased plasma-free fatty acids, insulin resistance, oxidative stress, and chronic inflammation, and its pathophysiological pathways include inflammation, diabetes, hypertension, CVDs, and obesity.

**Figure 1:**
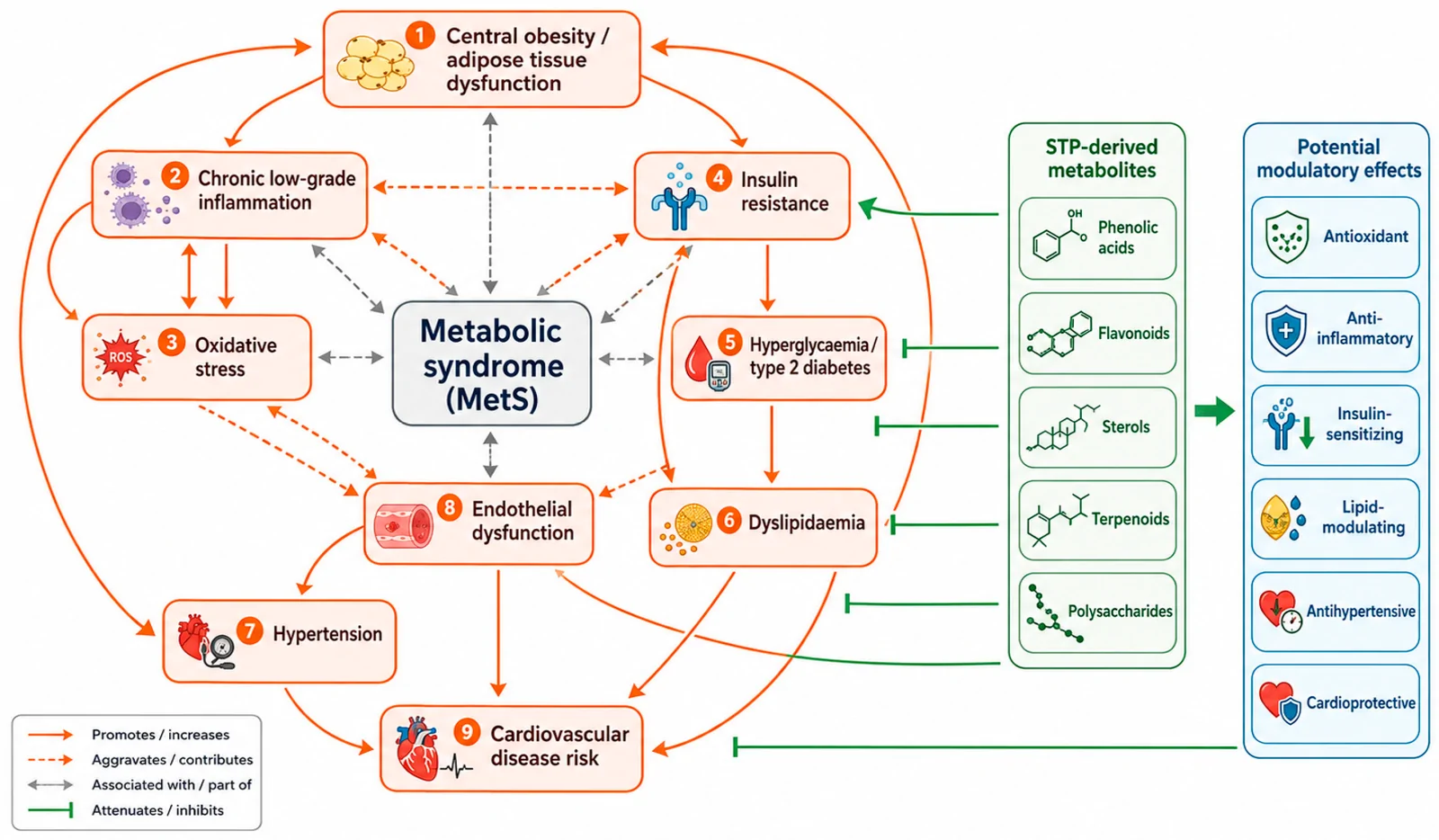
Conceptual representation of the interrelationships among the main components of metabolic syndrome (MetS) and the potential modulatory effects of salt-tolerant plant (STP)-derived metabolites. The figure was conceptually designed by the authors as a schematic synthesis based on the literature reviewed in this manuscript and prepared with AI-assisted graphical support, followed by author revision.

Although the MetS-related disorders discussed below are presented in separate subsections for clarity, they should be understood as biologically interconnected processes rather than independent conditions. Central obesity promotes adipose tissue dysfunction and chronic low-grade inflammation, which contribute to oxidative stress and impaired insulin signaling. Insulin resistance, in turn, favors hyperglycemia, altered lipid metabolism, increased circulating free fatty acids, and an atherogenic lipid profile, while inflammation and oxidative stress further aggravate endothelial dysfunction and blood pressure regulation. Together, these alterations reinforce each other and increase the risk of T2DM and CVDs. In this context, STP-derived metabolites may be relevant because they can potentially modulate overlapping mechanisms involved in MetS, such as oxidative stress, inflammatory signaling, glucose metabolism, lipid homeostasis, endothelial function, and cardiometabolic regulation (Figure 1). Therefore, the following sections are organized according to the main MetS-associated outcomes reported in the literature, while recognizing that these outcomes are mechanistically interconnected.

### 1.2. Inflammation

Inflammation is a defense mechanism consisting of a physiological response of the organism against, for example, invasion by pathogenic microorganisms. It can also be triggered by tissue injury, cell death, cancer, ischemia, and degeneration [24]. During inflammation, pro- and anti-inflammatory mediators are released, although some mediators, such as interleukin (IL)-12, exhibit pro- and anti-inflammatory behavior [25]. Several inflammatory mediators, including cytokines [e.g., interferons, interleukins and tumor necrosis factor α (TNF)-α], chemokines (e.g., monocyte chemoattractant protein 1), eicosanoids (e.g., prostaglandins and leukotrienes) and the potent inflammation-modulating transcription factor nuclear factor κB (NF-κB), have been widely studied in association with several human diseases. Chronic low-grade inflammation is a hallmark of MetS, driven primarily by adipose tissue dysfunction. As visceral fat accumulates, it acts as an endocrine organ, releasing pro-inflammatory cytokines, including TNF-α, interleukin-6 (IL-6), and C-reactive protein (CRP), which contribute to systemic inflammation and exacerbate metabolic dysregulation. One key aspect of this inflammatory process is the effect of adipokines (hormones produced by adipose tissue) that influence insulin sensitivity. For instance, elevated levels of TNF-α and IL-6 impair insulin-signaling pathways, promoting insulin resistance, a central feature of MetS. Insulin resistance, in turn, leads to elevated blood glucose levels, increased free fatty acid release, and the production of advanced glycation end-products (AGEs), all of which fuel further inflammation and oxidative stress. This creates a vicious cycle where inflammation and metabolic dysfunction perpetuate each other. Additionally, inflammation is closely linked to dyslipidemia, another component of MetS. Pro-inflammatory cytokines promote the overproduction of very low-density lipoprotein (VLDL) and decrease high-density lipoprotein (HDL) levels, leading to an atherogenic lipid profile. This lipid imbalance contributes to the development of atherosclerosis, a major risk factor for cardiovascular diseases associated with MetS. Hypertension is also exacerbated by inflammation, as inflammatory cytokines increase oxidative stress and reduce nitric oxide (NO) availability, leading to endothelial dysfunction and vasoconstriction. This contributes to elevated blood pressure, further complicating the cardiovascular risks associated with MetS.

### 1.3. Diabetes Mellitus (DM)

DM refers to a group of autoimmune, metabolic, and genetic disorders sharing hyperglycemia as the common feature, which can lead to microvascular problems in the retina, kidney, or peripheral nerves [26]. DM results from defects in insulin secretion, insulin action, or both, involving β-cell dysfunction, insulin resistance, impaired hepatic glucose regulation, reduced peripheral glucose uptake and, in some cases, increased intestinal glucose absorption through carbohydrate-hydrolyzing enzymes such as α-glucosidase and α-amylase [27]. It is estimated that approximately 588 million people (aged 20–79 years) are diabetic, and the prediction for 2050 is a huge increase in this number, with approximately 852 million people [28]. This health problem can be palliated through the administration of conventional drugs to patients such as acarbose, miglitol, and voglibose, which have been clinically tested as efficient inhibitors of α-glucosidase and α-amylase activities [29,30]. Nevertheless, these drugs have several side effects such as abdominal distension, flatulence, and meteorism [31]. Insulin resistance is a key factor in the pathogenesis of type 2 diabetes and plays a central role in MetS [27]. It occurs when the body’s cells become less responsive to insulin, leading to elevated blood glucose levels. Over time, this compensatory mechanism fails, resulting in persistent hyperglycemia and the onset of T2DM. Chronic insulin resistance is also associated with increased production of inflammatory cytokines, such as TNF-α, which exacerbates metabolic dysfunction. Insulin resistance is the foundation upon which other conditions, such as hypertension and dyslipidemia, build [27]. Insulin resistance promotes the storage of visceral fat, leading to obesity, which further worsens insulin sensitivity [32]. Additionally, insulin resistance contributes to dyslipidemia by increasing free fatty acid transport, which leads to elevated triglyceride and low-density lipoprotein (LDL) levels [32]. These lipid abnormalities, in turn, exacerbate cardiovascular risk, as described above. Thus, managing insulin resistance is key to addressing the broader spectrum of MetS and preventing its complications, including diabetes and cardiovascular diseases [32].

### 1.4. Hypertension

Hypertension, or elevated blood pressure, is one of the most significant risk factors for CVDs, such as coronary artery disease, stroke, and heart failure. Hypertension develops when the force of blood against artery walls remains consistently high, leading to increased stress on the vascular system, promoting endothelial dysfunction, arterial stiffness, and atherosclerosis [33]. Prolonged high blood pressure can result in severe damage to blood vessels, increasing the likelihood of heart attacks, strokes, and other cardiovascular complications [33]. Hypertension is both a component and a consequence of MetS. The interplay between obesity, insulin resistance, and hypertension is particularly strong, as obesity often triggers an overactivation of the renin–angiotensin system (RAS), which increases blood pressure. Additionally, insulin resistance can impair endothelial function by reducing NO bioavailability, which promotes vasoconstriction, further elevating blood pressure. This bidirectional relationship between hypertension and metabolic disturbances underscores the need for integrated therapeutic approaches that target both blood pressure control and underlying metabolic disorders [34,35].

### 1.5. Cardiovascular Diseases (CVDs)

CVDs encompass a range of conditions affecting the heart and blood vessels, including coronary artery disease, heart failure, and stroke. These diseases are often driven by the buildup of atherosclerotic plaques in the arteries, which restricts blood flow and increases the risk of blood clots. Chronic inflammation and oxidative stress are common features of CVDs, contributing to the progression of arterial damage and plaque formation [36,37].

MetS significantly increases the risk of CVDs. The cluster of conditions associated with MetS, including hypertension, dyslipidemia, and central obesity, directly contributes to the development of atherosclerosis and other cardiovascular complications. Insulin resistance, a hallmark of MetS, further aggravates cardiovascular risk by disrupting normal lipid metabolism, leading to elevated levels of LDL and triglycerides. These lipid abnormalities promote the formation of fatty deposits in arteries, accelerating atherosclerosis and increasing the risk of heart attacks and strokes. Addressing CVDs in patients with MetS requires management of the underlying metabolic abnormalities alongside traditional cardiovascular risk factors [36,37].

### 1.6. Obesity

Obesity, particularly central or visceral obesity, is a primary component of MetS. It is characterized by excessive fat accumulation, especially around the abdominal area, which significantly increases the risk of insulin resistance, hypertension, and dyslipidemia. The adipose tissue in obesity is not simply a fat storage site but an active endocrine organ that secretes various pro-inflammatory cytokines such as IL-6 and TNF-α, which drive chronic inflammation and metabolic dysfunction. Obesity contributes to the development of both insulin resistance and hypertension. The chronic low-grade inflammation caused by adipose tissue dysregulation exacerbates insulin resistance by interfering with insulin signaling pathways. Furthermore, the activation of the renin–angiotensin system (RAS) in obese individuals contributes to increased blood pressure. Together, these factors form a vicious cycle, where obesity perpetuates metabolic abnormalities that further contribute to the onset of diabetes and cardiovascular diseases. Thus, effective management of MetS requires strategies aimed at reducing obesity through lifestyle modifications and targeted therapeutic interventions [38].

Dyslipidemia refers to abnormal levels of lipids in the blood, including elevated low-density LDL cholesterol, high triglycerides, and low levels of HDL cholesterol. Dyslipidemia is a key component of MetS and a major contributor to the development of atherosclerosis. The imbalance in lipid levels accelerates the deposition of fatty plaques within arterial walls, increasing the risk of coronary artery disease, stroke, and peripheral vascular disease. In MetS, dyslipidemia is closely associated with insulin resistance. Insulin resistance alters lipid metabolism by increasing the release of free fatty acids from adipose tissue, which, in turn, leads to elevated triglycerides and LDL cholesterol levels. These lipid abnormalities further contribute to cardiovascular risk by promoting plaque formation and inflammation within arteries. Therefore, treating dyslipidemia in the context of metabolic syndrome involves managing both insulin resistance and abnormal lipid levels to prevent long-term cardiovascular complications [36,39].

### 1.7. Available Management of MetS

Conventional MetS treatments include modifications in lifestyle (e.g., increased physical activity, healthier eating habits) and pharmacological agents to reduce blood pressure and the levels of glucose and triglycerides [2,40]. However, such drugs often have reduced efficacy and exhibit undesirable side effects such as flatulence. Other strategies are acknowledged as promising for the management of MetS and include, for example, the Mediterranean diet and the use of nutraceuticals such as plant extracts, spices, herbs, and essential oil extracts from, for example, *Curcuma longa* (turmeric), *Allium sativum* (garlic), and *Vitis vinifera* (grapes) [2]. Although presenting benefits for preventing and managing MetS, the use of such natural products is still under investigation, and they cannot yet be considered a real alternative to pharmacotherapy [2]. However, the use of nutraceuticals in the management of MetS may be a promising field in the development of novel therapies [21,41].

### 1.8. Objectives and Methodology

The objective of this review is to provide an integrated and critical overview of the potential of STPs as sources of bioactive compounds for the prevention and management of MetS and its main associated conditions, namely chronic inflammation, T2DM, hypertension, cardiovascular disorders, dyslipidemia and obesity.

This review was designed as a comprehensive narrative review. Therefore, it was not intended to be a strict systematic review or meta-analysis. Nevertheless, the literature search, screening and selection process followed the main principles of the PRISMA approach, where applicable, to improve transparency and reproducibility. PRISMA guidelines were used as a framework to describe the search strategy, eligibility criteria, exclusion criteria, duplicate removal, screening process, data extraction and qualitative evaluation of the evidence.

A comprehensive literature search was performed using PubMed, Web of Science, Embase, and SciFinder, as well as Google Scholar as a supplementary search engine. The search covered all available literature published up to 2026, without an initial restriction on publication year, in order to include both early ethnomedicinal records and the most recent phytochemical and pharmacological studies. Google Scholar was used to identify additional relevant studies, especially ethnobotanical, phytochemical and older pharmacological reports that may not be fully indexed in the other databases.

The search strategy combined terms related to plant ecology, traditional use, phytochemistry and MetS-associated bioactivities. The main search strings included combinations of the following terms: (“halophyte” OR “salt-tolerant plant” OR “salt tolerant plant”) AND (“metabolic syndrome” OR “diabetes” OR “type 2 diabetes” OR “antidiabetic” OR “anti-diabetic” OR “inflammation” OR “anti-inflammatory” OR “anti-inflammatory” OR “hypertension” OR “cardiovascular” OR “cardioprotection” OR “dyslipidaemia” OR “lipid metabolism” OR “obesity” OR “anti-obesity” OR “antioxidant”) AND (“ethnomedicinal” OR “ethnopharmacology” OR “traditional medicine” OR “phytochemistry” OR “bioactive compounds” OR “bioactivity”). Additional searches were performed using the scientific names of selected species identified during the screening process, combined with terms such as “extract”, “phenolics”, “flavonoids”, “sterols”, “terpenes”, “*in vitro*”, “*in vivo*”, “toxicity” and “mechanism”.

Search results were exported when possible and manually checked to remove duplicates. Approximately 900 records were initially retrieved from the database and supplementary searches. After duplicate removal, around 600 records were screened by title and abstract for relevance to halophytes/STP, ethnomedicinal use, phytochemistry and/or MetS-related bioactivities. Records clearly unrelated to the topic were excluded at this stage. Approximately 300 full-text articles were then assessed for eligibility, and around 250 studies were considered relevant for inclusion in the final qualitative synthesis. These numbers are approximate because, in addition to database searches, the review included iterative searches using the scientific names of relevant species and supplementary manual screening of references.

Eligible studies included original research articles, reviews, ethnobotanical reports, and experimental studies reporting ethnomedicinal uses, phytochemical composition, and/or biological activities of halophytes and STPs relevant to MetS or its associated pathological conditions. Inclusion criteria were: (i) full-text articles written in English; (ii) clear identification of the plant species; (iii) evidence that the species is halophytic or salt-tolerant; (iv) information related to ethnomedicinal use, phytochemical composition and/or biological activity; and (v) relevance to at least one MetS-associated condition, including oxidative stress, inflammation, diabetes, obesity, dyslipidaemia, hypertension or cardiovascular dysfunction.

Studies were excluded when they lacked sufficient taxonomic information, when the plant species could not be confirmed as halophytic or salt-tolerant, when the reported biological activity was unrelated to MetS-associated conditions, or when the experimental design was insufficiently described. Non-English publications, articles without accessible full text, conference abstracts, patents, and studies focused exclusively on agronomic, ecological, or nutritional aspects without relevant phytochemical or bioactivity data were not included in the main analysis. However, some ecological or taxonomic sources were considered when necessary to contextualize species distribution, salinity tolerance, or traditional use.

Plant nomenclature and accepted scientific names were checked using the World Flora Online [42]. The halophytic or salt-tolerant nature of each species was verified, whenever possible, using the eHALOPH database, accessed in 2026. When a species was not listed in eHALOPH, additional scientific literature was consulted to confirm whether it naturally occurs in saline, coastal, estuarine, salt marsh, desert, mangrove or other salt-affected environments.

Data extraction was performed manually using a structured approach. For each relevant study, the following information was collected when available: plant species, botanical family, plant part used, traditional or ethnomedicinal use, extraction solvent or method, extract type, major reported compounds or compound classes, experimental model, assay conditions, tested concentrations or doses, main biological effects, proposed mechanisms of action and relevance to MetS-related conditions.

The quality and strength of the evidence were assessed qualitatively. Ethnomedicinal uses were considered supportive background evidence, especially when consistent with experimentally reported activities. *In vitro* studies were interpreted as preliminary evidence and evaluated according to assay relevance, concentration range, reproducibility, cytotoxicity/selectivity, use of controls and consistency with phytochemical data. *In vivo* studies were considered stronger evidence, particularly when they included appropriate controls, dose–response evaluation and endpoints directly related to MetS, such as glycemia, lipid profile, inflammatory markers, oxidative stress biomarkers, body weight, adiposity, blood pressure or cardiovascular function. Studies involving isolated compounds, mechanistic assays or molecular targets were used to support the interpretation of possible modes of action.

To strengthen the critical analysis, species, extracts and compounds were prioritized according to the convergence of evidence from different sources, including traditional use, phytochemical characterization, reproducible bioactivity, relevance of the experimental model, and the presence of compounds previously associated with anti-inflammatory, antidiabetic, antioxidant, cardioprotective, lipid-lowering or anti-obesity effects. Greater weight was given to studies combining phytochemical characterization with biologically relevant *in vitro* or *in vivo* models. Exploratory *in vitro* findings were clearly distinguished from stronger preclinical evidence, and the main knowledge gaps were identified, particularly regarding safety, toxicity, bioavailability, extract standardization, mechanisms of action, clinical validation and sustainable use of plant biomass.

## 2. Halophytes and Other STPs as Sources of Health Improvement Commodities

Halophytes, together with other STPs, represent the characteristic flora of saline environments, particularly marine-influenced coastal ecosystems such as beaches, dunes, rocky shores, salt marshes, estuaries, and tidal or supratidal zones, as well as inland saline or arid soils. These conditions cause the production and accumulation of reactive oxygen species (ROS) in the plants, in turn leading to oxidative stress, cellular and tissue damage, metabolic disorders, and senescence processes [43,44,45]. To manage such oxidative stress conditions, those plants are equipped with highly effective antioxidant defense systems, such as antioxidant enzymatic mechanisms (e.g., superoxide dismutases and glutathione peroxidase) and synthesis and accumulation of secondary metabolites (e.g., polyphenols, terpenoids, alkaloids) that counteract ROS production and inhibit oxidative chain reactions [6,46]. Besides their important protective role in the plant, such molecules show important bioactivities highly relevant for human health improvement, including antioxidant, anti-inflammatory, and antidiabetic, just to name a few. Such bioactivities lead to beneficial therapeutic properties linked to the use of some species in traditional medicine (e.g., *Carpobrotus edulis* L.), or as food (e.g., *Chenopodium quinoa* Willd, *Salicornia* spp.). It confers halophytes and other STPs a high potential to provide nutraceuticals, functional ingredients, standardized extracts, or bioactive preparations with health-promoting properties to address different pathologies, including those associated with MetS.

Moreover, halophytes and other STPs have an important advantage over common glycophyte cash crops: they can be cultivated in diverse saline production systems, such as those using underutilized saline soils and irrigation water. In fact, while most of the crop species used in traditional agriculture are salt sensitive, presenting approx. a 10% yield decrease as the soil salinity increases over the 4–8 dS/m range, the growth of several STPs is stimulated within a salinity range of 15–25 dS/m [47,48]. This is of extreme importance in the actual context of soil and water salinization, which is a global problem since salt-affected areas are increasing day by day because of scanty rainfall, water contamination, and poor irrigation systems [12,49]. The use of halophytes and other STPs with economic potential is considered a valuable strategy to exploit and bioremediate saline soils and/or waters, aiming for sustainable water management and soil conservation when establishing cost-efficient and environmentally friendly agro-ecosystems [48,50].

## 3. Ethnomedicinal Uses of Halophytes and Other STPs Related to MetS Management

Researchers have long been compiling and categorizing the ethnomedicinal uses of plants in different areas of the world, including the Mediterranean region [51,52], North Africa [53], and the North Sea [54]. However, studies focusing on the ethnomedicinal uses of STP are infrequent [6,7,8,10,15].

Previous reviews have highlighted the broad ethnomedicinal relevance of halophytes and other STPs, documenting their traditional use across different geographical regions and plant families. For example, Ksouri et al. [6] provided a comprehensive overview of folkloric medicinal uses, reported bioactivities and major chemical constituents of several halophytic species, while Qasim et al. [8] focused on coastal halophytes from the Arabian Sea region, and Al-Oudat and Qadir [15] surveyed the traditional uses of halophytic flora from Syria. Collectively, these studies show that STPs have been traditionally used for a wide range of purposes, including the treatment of inflammatory, infectious, digestive, respiratory, metabolic and skin-related disorders, generally using the whole plant or specific organs such as roots, leaves, flowers, fruits or seeds, prepared as infusions, decoctions, tinctures, cataplasms, or consumed fresh, dried or cooked [6,8,15]. Building on this background, the present review focuses specifically on STPs traditionally used or investigated for conditions associated with metabolic syndrome, namely diabetes, obesity, hypertension, cardiovascular disorders and inflammation.

Table 1 summarizes several STPs and their traditional medicinal uses, relevant for the treatment of the following MetS-related conditions: chronic inflammation, diabetes, hypertension, cardiovascular disorders, and obesity. In brief, in the Mediterranean area, species such as *Aerva javanica* (Burm. F.) Juss. ex J.A. Schultes, *Salicornia ramosissima* J. Woods, *Sarcocornia fruticosa* (L.) A.J. Scott, and *Tamarix gallica* L. have traditional uses towards obesity, while *Atriplex halimus*, *A. littoralis*, *Halimione portulacoides*, *Salicornia herbacea J.Woods*, *Sarcocornia fruticosa* (L.) A.J. Scott, *Salsola kali* L., *Suaeda vera* Forssk. ex J.F. Gmel., *Artemisia maritima* L., *Tripolium pannonicum* (Jacq.) Dobrocz, *Raphanus raphanistrum* L., *Ipomoea pes-caprae* (Linn.) R. Br., *Citrullus colocynthis* L., *Prosopis farcta* (Banks and Sol) Macbr, *Juncus maritimus* Lam., *Cynodon dactylon* (L.) Pers., *Elymus repens* (L.) Gould, *Rhizophora mangle* L., and *Zygophyllum album* L. are used to treat diabetes. The following species are used in the treatment of cardiovascular and vascular problems: *Salsola imbricata* Forssk., *S. kali* L., *S. soda* L., *Hyphaene thebaica* (L.) Mart., *Artemisia campestris* L. ssp *maritima* Arcangeli., *A. scoparia* Waldst. & Kit., *Dittrichia viscosa* (L.) Greuter, *Ipomoea pes-caprae* (Linn.) R. Br., *Schenkia spicata* (L.) Fritsch., *Arundo donax* L., *Cynodon dactylon* (L.) Pers., *Portulaca olearacea* L., *Peganum harmala* L., *Tribulus terrestris* L., *and Zygophyllum coccineum* L.

Halophytic species and STPs alleviating inflammatory conditions include *Atriplex prostrata* Boucher ex DC., *Crithmum maritimum* L., *Artemisia capillaris* Thun., *A. scoparia* Waldst. & Kit., *Helichrysum italicum* (Roth) G.Don, *Cleome brachycarpa* Vahl. ex DC., *Ipomoea pes-caprae* (L.) R. Br., *Plantago major* L., *Armeria maritima* (Mill.) Willd., *Cymbopogon jwarancusa* (Jones) Schult., *and Elymus repens* (L.) Gould Boiss. From Table 1, it can be noted that *Artemisia* species generally have multiple medicinal uses, including inflammation, hypertension, and diabetes. Similarly, *Ipomoea pes-caprae* is traditionally used to treat hypertension, diabetes, and inflammation.

Several species included in Table 1 are recognized in official pharmacopeias, supporting their long-standing medicinal relevance and providing quality standards for specific herbal drugs. Among them, *Plantago lanceolata* L. is described in the European Pharmacopeia as *Plantaginis lanceolatae folium* (ribwort plantain leaf; Ph. Eur. 01/2008:1884 [55]), while *Elymus repens* (L.) Gould, under the pharmacopeial synonym *Agropyron repens*, is included as *Agropyri repentis rhizoma* or *Graminis rhizoma* (couch grass rhizome; Ph. Eur. 04/2011:1306 [56]). *Verbena officinalis* L. is also recognized as *Verbenae herba* (aerial parts) in the European Pharmacopeia and has additionally been included in the Chinese Pharmacopeia. Other species are represented in non-European pharmacopeias, particularly Asian and traditional medicine compendia. For example, *Artemisia scoparia* Waldst. and Kitam. is listed in the Chinese Pharmacopeia as *Artemisiae scopariae herba* (aerial parts), *Chrysanthemum indicum*, as *Chrysanthemi indici flos* (dried inflorescence), *Portulaca oleracea* L. as *Portulacae herba* (dried aerial parts), *Tribulus terrestris* L. as *Tribuli fructus* (dried fruit), and *Bassia scoparia* (L.) Voss. as *Kochiae fructus* (dried ripe fruit). In the Ayurvedic Pharmacopeia of India, *Citrullus colocynthis* (L.) Schrad. is included as *Indravaruni*, *Cynodon dactylon* (L.) Pers. as *Durva* (whole plant), and *Daemia cordata* L., through the related pharmacopoeial taxon *Pergularia daemia*, as *Visanika*. In addition, *Tamarix gallica* L. is listed in the Unani Pharmacopeia of India as *Mayeen Kalan* (dried galls), whereas *Visnaga daucoides* Gaertn. (=*Ammi visnaga* (L.) Lam.) is pharmacopeially referenced for its fruits, including in relation to the Egyptian Pharmacopeia. Overall, the pharmacopeial inclusion of these species, or their accepted synonyms, highlights their relevance as medicinal plants and provides a regulatory and quality-control framework for selected plant parts. However, for most species covered in this review, no specific monographs were identified in the Portuguese Pharmacopeia, and, within the European context, the European Pharmacopeia remains the most relevant official reference.

**Table 1:** Examples of ethnomedicinal halophytes and other salt-tolerant plants, used in different countries/regions of the world and their therapeutic applications, relevant for metabolic syndrome-related conditions (inflammation, diabetes, hypertension, cardiovascular disorders, and obesity).

Plant Family	Species	Plant Organs/ Administration	Country/Region	References
Inflammation				
Aizoaceae	*Mesembryanthemum crystallinum* L.	Leaves	Algeria (North Africa/Mediterranean region)	[57]
	*Mesembryanthemum nodiflorum* L.	Leaves	Algeria (North Africa/Mediterranean region)	[57]
	*Tetragonia tetragonoides* (Pall.) Kuntze	Leaves	New Zealand, Australia, Japan (Oceania), the Republic of Korea (East Asia)	[58]
Amaranthaceae	*Arthrocaulon macrostachyum* (Moric.) Piirainen & G.Kadereit	Leaves, seeds	Algeria (North Africa/Mediterranean region)	[57]
	*Atriplex prostrata* Boucher ex DC.	Whole plant	Algeria (North Africa/Mediterranean region), Bulgaria (Southeastern Europe)	[57]
	*Salicornia europea* L. (=*Salicornia herbacea* L.)	Aerial parts	Korea (East Asia)	[56,59]
	*Suaeda maritima* (L.) Dumort	Extract	Algeria (North Africa/Mediterranean region),	[57]
Apiaceae	*Crithmum maritimum* L.	Aerial organs Decoctions	Italy (Mediterranean region)	[60]
Asteraceae	*Helichrysum italicum* (Roth) G.Don	Leaves and flowers Infusions, decoctions, essential oils	Italy, Spain (Mediterranean region) Portugal (Mediterranean–Atlantic region)	[60,61]
	*Pluchea indica* (L.) Less.	Roots	India (South Asia)	[62]
	*Limbarda crithmoides* (L.) Dumort.	Leaves	Algeria (North Africa)	[57]
Cleomaceae	*Cleome brachycarpa* Vahl. Ex DC	Whole plant	Pakistan (South Asia)	[63]
Caryophyllaceae	*Spergularia marina* (L.) Besser	Extract	Algeria (North Africa/Mediterranean region)	[57]
Convolvulaceae	*Ipomoea pes-caprae* (L.) R.Br.	All plant Decoctions	Algeria (North Africa/Mediterranean region)	[57]
	*Cressa cretica* L.	Leaves	Algeria (North Africa/Mediterranean region)	[57]
Frankeniaceae	*Frankenia pulverulenta* L.	Leaves	Algeria (North Africa/Mediterranean region)	[57]
Juncaceae	*Juncus acutus* L.	Leaves	Algeria (North Africa/Mediterranean region)	[57]
	*Peganum harmala* L.	Fruits, seeds	Algeria (North Africa/Mediterranean region), Egypt (North Africa/Eastern Mediterranean), Iran (West Asia)	[51,57,64]
Plantaginaceae	*Plantago lanceolata* L. *	Leaves powder	Turkey (Eastern Mediterranean/West Asia), Pakistan (South Asia)	[65]
	*P. major*	Leaves n.a.	Turkey (Eastern Mediterranean/West Asia)	[65]
Plumbaginaceae	*Armeria maritima* (Mill.) Willd.	Aerial parts	Afghanistan (Central–South Asia), Portugal (Mediterranean–Atlantic region)	[66,67]
	*Limonium delicatulum* (Girard) Kuntze	Seeds	Algeria (North Africa/Mediterranean region)	[57]
Poaceae	*Cymbopogon iwarancusa* (Jones ex Roxb.) Schult.	Whole plant	Pakistan (South Asia)	[63,68]
	*Elymus repens* (L.) Gould *	n.a. Ointment	Serbia (Southeastern Europe)	[69]
Tamaricaceae	*Tamarix africana* Poir.	Leaves	Algeria (North Africa/Mediterranean region)	[57]
Verbenaceae	*Verbena officinalis* L. *	Leaves Topical application	Spain (Mediterranean region)	[70]
	*Zygophyllum coccineum* L.		Egypt (North Africa/Eastern Mediterranean), Jordan (West Asia/Middle East)	[51,71]
Diabetes				
Amaranthaceae	*Anabasis articulata* (Forssk.) Moq	Leaves Decoction	Algeria (North Africa/Mediterranean region), Libya (North Africa/Mediterranean region)	[72]
	*Atriplex halimus* L.	Roots, whole plant, leaves	Algeria (North Africa/Mediterranean region), Poland (Central Europe), Israel (West Asia/Eastern Mediterranean)	[73,74]
	*A. littoralis* L.	Fruits, leaves, shoots	Algeria (North Africa/Mediterranean region), Italy (Mediterranean region), Lesotho (Southern Africa)	[57]
	*Chenopodium quinoa* Willd.	Seeds	Morocco (North Africa/Mediterranean–Atlantic region)	[75]
	*Gomphrena globosa* L.	Leaves and flowers	Brazil (South America), Trinidad and Tobago (Caribbean region)	[76]
	*S. europaea* L. (=*S. herbacea*)	Branches and leaves	Portugal (Mediterranean–Atlantic region), Korea (East Asia)	[77,78]
	*Salicornia fruticosa* (L.) (=*Sarcocornia fruticosa* (L.) A.J.Scott)	Branches, leaves, seeds	Portugal (Mediterranean–Atlantic region)	[79]
	*Salsola kali* L.	Roots, whole plant	Poland (central Europe)	[80]
	*Suaeda vera* Forssk. ex J.F.Gmel.	Whole plant, seeds	Morocco (North Africa/Mediterranean–Atlantic region)	[81]
Apiaceae	*Pituranthos scoparius* Deverra scoparia Coss. & Durieu	Leaves, flowers	Tunisia (North Africa/Mediterranean region)	[82]
Apocynaceae	*Daemia cordata* L. (=*Pergularia tomentosa* L.) *	n.a.	Tunisia (North Africa/Mediterranean region)	[82,83]
Asparagaceae	*Artemisia maritima* L.	n.a.	North Sea (Northern Europe), India (South Asia)	[54,84]
	*Galatella tripolium* (L.) Galasso, Bartolucci & Ardenghi (=*Tripolium pannonicum* (Jacq.) Dobrocz.)	Leaves, roots	Italy (Mediterranean region)	[48,85]
Asteraceae	*P. indica*	Leaves Tea	Thailand (Southeast Asia)	[67,86,87]
Brassicaceae	*Raphanus raphanistrum* L.	Aerial part Boiled	Portugal (Mediterranean–Atlantic region)	[79,88]
Caryophyllaceae	*Paronychia argentea* Lam.	Whole plant	Jordan (West Asia/Middle East)	[71]
Convolvulaceae	*I. pes-caprae*	“Vriddhadaru” in Ayurvedic medicine	India (South Asia)	[89]
	*Cressa cretica* L.	Leaves	Algeria (North Africa/Mediterranean region)	[57,90]
Cucurbitaceae	*Citrullus colocynthis* (L.) Schrad. *	Fruits, roots	Pakistan (South Asia)	[63]
Fabaceae	*Prosopis farcta* (Banks & Sol.) J.F.Macbr	Fruits	Iran (West Asia)	[64]
Juncaceae	*Juncus maritimus* Lam.	Fruits, rhizome	Algeria (North Africa/Mediterranean region), Germany (Central Europe), Lesotho (Southern Africa)	[57,91]
Malvaceae	*Thespesia populnea* (L.) Sol. ex Corrêa	Fruits	India (South Asia)	[92,93,94]
Plumbaginaceae	*Armeria maritima* (Mill.) Willd.	Aerial parts	Afghanistan (Central–South Asia), Portugal (Mediterranean–Atlantic region)	[66]
Poaceae	*Cynodon dactylon* (L.) Pers. *	Whole plant Crushed leaves	India (South Asia)	[95]
	*Phragmites australis* (Cav.) Trin. ex Steud.	Leaves	Algeria (North Africa/Mediterranean region)	[57]
Rhizophoraceae	*Bruguiera gymnorhiza* (L.) Lam. ex Savigny	Fruits	Mauritius (Indian Ocean region)	[96]
	*Rhizophora mangle* L.	Fruits, leaves, whole plant	Pakistan (South Asia)	[97]
Tamaricaceae	*Tamarix gallica* L. *	Bark, leaves	Algeria (North Africa/Mediterranean region), Greece (Mediterranean region), Italy (Mediterranean region), Kuwait (West Asia/Arabian Peninsula), Portugal (Mediterranean–Atlantic region)	[57,98,99,100,101]
Zygophyllaceae	*Zygophyllum album* L.f.	n.a.	Tunisia (North Africa/Mediterranean region)	[6]
	*Zygophyllum coccineum* L.	n.a.	Egypt (North Africa/Eastern Mediterranean), Jordan (West Asia/Middle East)	[51,71]
Hypertension				
Amaranthaceae	*Gomphrena globosa* L.	Leaves and flowers	Brazil (South America), Trinidad and Tobago	[76,102]
Arecaceae	*Hyphaene thebaica* (L.) Mart.	Fruits Decoction	Egypt (North Africa/Eastern Mediterranean)	[53]
Asparagaceae	*Artemisia campestris* L. *subsp maritima*	Stems, leaves Infusions, decoctions	Portugal (Mediterranean–Atlantic region)	[103]
Asteraceae	*Chrysanthemum indicum* L. *	Flowers	Chinese traditional medicine (East Asia)	[104]
	*Artemisia scoparia* Waldst. & Kit. *	Aerial parts Tea	Asian countries Asian (including East Asia, South Asia, Southeast Asia and West Asia)	[105,106,107]
Convolvulaceae	*I. pes-caprae*	Leaves Infusions	n.a.	[89,108]
Nitrariaceae	*Nitraria retusa* (Forssk.) Asch.	Aerial parts	India (South Asia), Tibet/Himalayan region)	[109,110]
Poaceae	*C. dactylon* *	Whole plant	Tropics and subtropics regions	[111]
Zygophyllaceae	*Tribulus terrestris* L. *	n.a.	n.a.	[112]
	*Z. coccineum*	n.a.	Jordan (West Asia/Middle East)	[71]
Cardiovascular disorders				
Amaranthaceae	*A. halimus*	Leaves	Arab regions	[73,74,80]
	*Salsola kali* L.	n.a.	Cyprus (Eastern Mediterranean); Tunisia (North Africa/Mediterranean region), North Sea (Northern Europe)	[46,51,54]
	*Doda inermis Fourr* (=*Salsola soda* L.)	n.a.	Cyprus (Eastern Mediterranean)	[51]
Apiaceae	*Visnaga daucoides Gaertn.* (=*Ammi visnaga* (L.) Lam.)	n.a.	Algeria (North Africa/Mediterranean region), Egypt (North Africa/Eastern Mediterranean)	[51]
Asteraceae	*Dittrichia viscosa* (L.) Greuter	Leave, roots Topical application	Algeria (North Africa/Mediterranean region), Cyprus (Eastern Mediterranean), Spain (Mediterranean region), Italy (Mediterranean region), Portugal (Mediterranean–Atlantic region)	[51,60,88]
Gentianaceae	*Schenkia spicata* (L.) G.Mans. (=*Centaurium spicatum* (L.) Fritsch.)	n.a.	Egypt (North Africa/Eastern Mediterranean), Italy (Mediterranean region)	[51]
Malvaceae	*T. populnea*	Leaves	India (South Asia)	[92,93,94]
Nitrariaceae	*N. retusa*	Aerial parts	India (South Asia), Tibet (Himalayan region)	[109,110,113,114]
	*Peganum harmala* L.	Fruits and seed	Algeria (North Africa/Mediterranean region), Egypt (North Africa/Eastern Mediterranean), Iran (West Asia)	[51,64]
Poaceae	*Arundo donax* L.	Leaves, rhizome, roots	Italy (Mediterranean region)	[115]
Portulacaceae	*Portulaca oleracea* L. *	Roots, stems, leaves Boiling leaves’ vapor	Albania (Mediterranean region), Cyprus (Eastern Mediterranean), Iran (West Asia), Egypt (North Africa/Eastern Mediterranean)	[51,53,64]
Tamaricaceae	*T. gallica*	Bark, leaves	Algeria (North Africa/Mediterranean region), Greece (Mediterranean region), Italy (Mediterranean region), Kuwait (West Asia/Arabian Peninsula), Portugal (Mediterranean–Atlantic region)	[57,98,99,101]
Zygophyllaceae	*T. terrestris* *	Fruit	China (East Asia)	[112,116]
	*Z. coccineum*	n.a.	Algeria (North Africa/Mediterranean region), Egypt (North Africa/Eastern Mediterranean)	[51]
Obesity				
Amaranthaceae	*Bassia scoparia* (L.) Voss (=*Kochia scoparia* (L.) Schrad.)	Fruits	Japan (East Asia)	[117]
	*S. europaea* (=*S. herbacea*)	Fresh branches and leaves	Portugal (Mediterranean–Atlantic region), Korea (East Asia)	[77,78]
	*Salicornia ramosissima* (Hook.f.) J.Woods ex W.A.Clarke & E.S.Marshall (=*Salicornia europaea* L.)	Fresh branches and leaves	Portugal (Mediterranean–Atlantic region)	[79]
	*S. fruticosa* (=*S. fruticosa*)	Fresh branches, leaves, seeds	Portugal (Mediterranean–Atlantic region)	[79]
Asteraceae	*P. indica*	Leaves	Thailand (Southeast Asia)	[67]
Brassicaceae	*Lobularia maritima* (L.) Desv.	Aerial parts	Italy (Mediterranean region)	[118]
Malvaceae	*T. populnea*	Fruits	India (South Asia)	[92,93,94]
Nitrariaceae	*N. retus*	Aerial parts	India (South Asia), Tibet (Himalayan region)	[109,110,113,114]
Plumbaginaceae	*Armeria maritima* (Mill.) Willd.	Aerial parts	Afghanistan (Central–South Asia), Portugal (Mediterranean–Atlantic region)	[67]
Tamaricaceae	*T. gallica*	Bark, leaves	Algeria (North Africa/Mediterranean region), Greece (Mediterranean region), Italy (Mediterranean region), Kuwait (West Asia/Arabian Peninsula), Portugal (Mediterranean–Atlantic region)	[57,98,99,101]

n.a.: Data not available. Species marked with an asterisk (*) are included in official pharmacopeias, either under the currently accepted scientific name or under a pharmacopeial synonym.

**Figure 2:**
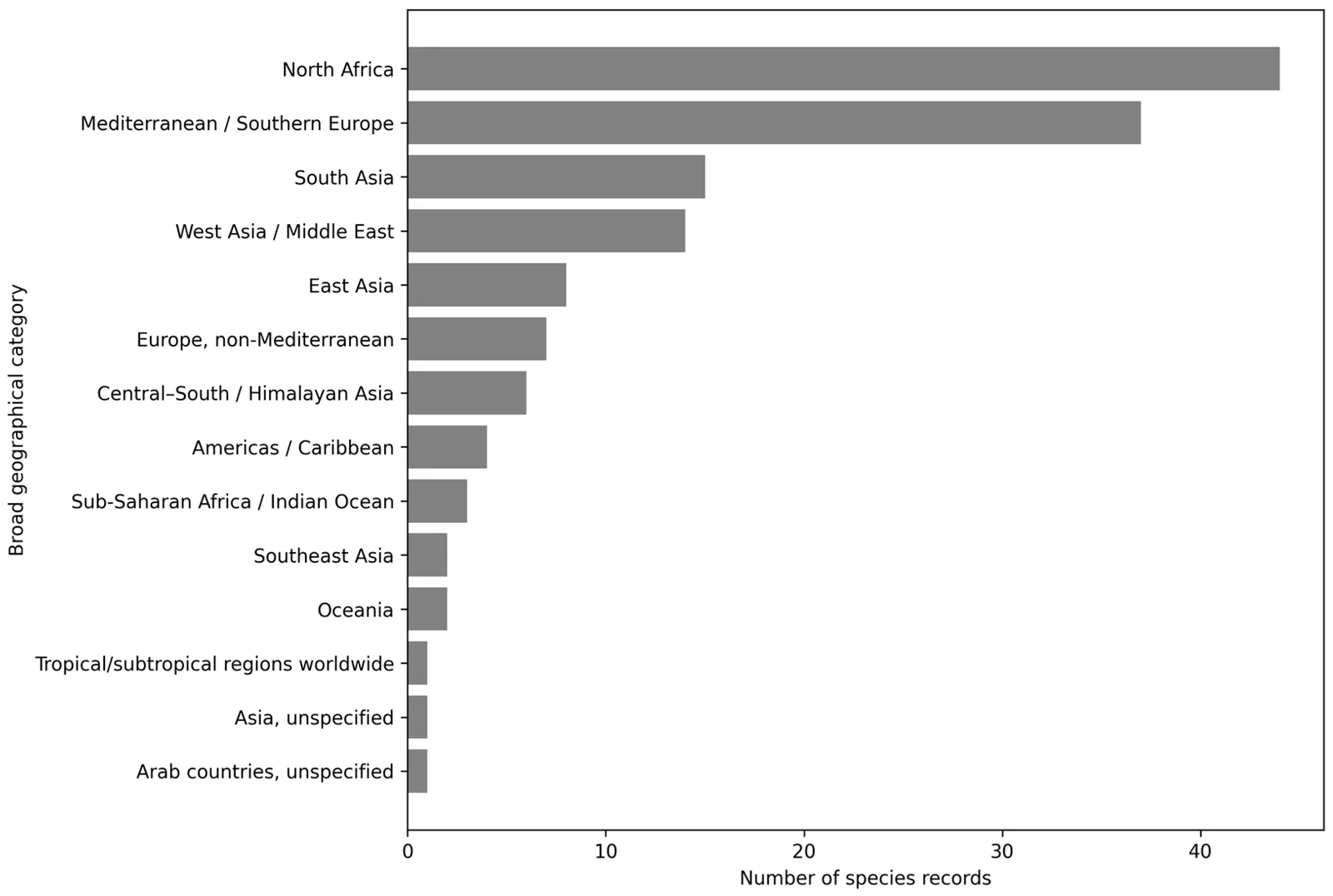
Geographical distribution of salt-tolerant plant records included in Table 1, grouped according to broad regions of traditional use. Records were counted by country or region of reported use; therefore, species reported from more than one country or region may contribute to more than one geographical category. Entries reported as not available were excluded from the graphical representation.

The geographical distribution of the species included in Table 1, summarized in Figure 2, shows a clear predominance of reports from North Africa and the Mediterranean/Southern European region, reflecting the strong ethnomedicinal relevance of salt-tolerant plants in Mediterranean and arid or semi-arid environments. South Asia and West Asia/Middle East also contributed a considerable number of records, highlighting the importance of these plants in traditional medical systems from Asian and Middle Eastern regions. In contrast, fewer records were found for East Asia, non-Mediterranean Europe, Oceania, the Americas/Caribbean and sub-Saharan Africa/Indian Ocean regions. Overall, this distribution indicates that the reviewed species are not restricted to the Mediterranean basin, but occur across a broad geographical range, supporting the global relevance of STPs as ethnomedicinal resources potentially associated with MetS.

The length of Table 1, which aimed at giving only some examples of medicinal halophytes and other STPs found in a few studies, demonstrates the importance and extensive uses of such plants in folk medicine. Herbal medicine has always had a fundamental role in human welfare, and it is still of utmost importance in many cultures today. In this sense, the ethnopharmacological knowledge of populations can serve as a basis to search for novel bioactive compounds to manage MetS [119,120,121]. Considering this, scientific research has explored and produced numerous studies about medicinal plants’ biological properties and chemical constituents, not only to advance plant drug discovery but also to validate the plants’ traditional uses [122]. However, for species with folk uses, research on their bioactivities and subsequent evidence supporting their medicinal claim is still scarce for many of those plants. Some information related to the biological properties of halophytes and other STPs related to MetS prevention can be found in the next section.

## 4. Biological Properties of STPs Related to MetS Prevention

The biological properties of halophytes and other STP relevant to MetS prevention are summarized in Table 2, highlighting their reported effects on key MetS-related conditions, including inflammation, diabetes, hypertension, cardiovascular disorders, and obesity.

### 4.1. Inflammation

Species with ethnomedicinal uses for inflammation have been validated in both *in vivo* and *in vitro* studies (Table 2). For instance, *Chenopodium album* essential oil significantly reduced ear edema in mice in a 12-*O*-tetradecanoylphorbol-13-acetate-induced inflammation model, with its anti-inflammatory effects attributed to α-pinene and linalool [124]. Similarly, *Cynodon dactylon* aqueous extracts reduced paw edema in rats, demonstrating its potential to inhibit inflammatory processes, attributed to glycosides and flavonoids [133]. Likewise, the *Salsola cyclophylla* aqueous–ethanolic extract (95%), with high amounts of phenolics and flavonoids, exhibited expressive anti-inflammatory capacity in reducing paw edema in rats after a 500 mg/kg/day p.o. administration [157].

Additionally, *in vitro* studies have revealed several species with promising anti-inflammatory properties, even in species without documented ethnomedicinal uses. *Salicornia herbacea* methanol extracts, for example, significantly suppressed NO production and pro-inflammatory cytokine expression in LPS-stimulated RAW 264.7 macrophages; these effects were due to isorhamnetin-3-*O*-beta-D-glucoside [126]. *Limonium spathulatum* methanol extracts inhibited NO production by 86.2% at 100 µg/mL, supported by its high total phenolic and flavonoid contents [132].

Other species with no ethnomedicinal claims have shown relevant anti-inflammatory activity *in vitro*. For instance, *Polygonum maritimum* dichloromethane extracts demonstrated potent inhibition of NO production (IC_50_ = 48 µg/mL) in LPS-stimulated macrophages, attributed to β-sitosterol, stigmasterol, and linolenic acid [130]. *Halimione portulacoides* chloroform extracts reduced NO production in macrophages with an IC_50_ of 109 µg/mL, likely due to moderate phenolic content [130]. *Carpobrotus edulis* acetone and water extracts inhibited α-15-lipoxygenase (IC_50_ = 22.3 µg/mL and 59.8 µg/mL, respectively), which is a key enzyme in the inflammatory process, probably linked to the high content of the extracts in phenolics and flavonoids [123]. Even lesser-known species have shown high potential. Linoleic acid obtained from an ethyl acetate-soluble fraction of *Asparagus officinalis* methanol extract inhibited COX-1 and COX-2 enzymes, with IC_50_ values of 14.6 µg/mL and 0.53 mg/mL [128]. Additionally, enzyme-treated asparagus leaf extracts blocked NO release and cytokine production in IL-1β-stimulated rat hepatocytes, indicating a broad anti-inflammatory activity [129]. Further exploration of STPs without traditional uses, such as methanol extracts from *Suaeda fruticosa* shoots, which reduced NO by 66.4% in LPS-stimulated macrophages, and a hexane extract from *Plantago coronopus* leaves, which exhibited NO inhibition with an IC_50_ of 98 µg/mL despite its low phenolic content, highlights the untapped potential of these species [127,130]. These findings collectively suggest that STP, both with and without ethnomedicinal backgrounds, offer a diverse and promising source of bioactive compounds for managing inflammatory conditions. Further research to isolate and characterize these bioactive constituents could pave the way for novel anti-inflammatory drugs.

### 4.2. Diabetes

Species with documented ethnomedicinal antidiabetic uses, supported by *in vivo* evidence, include, for example, *Anabasis articulata*, *Salicornia herbacea*, *Cressa cretica*, *Pluchea indica,* and *Atriplex halimus*. Decoctions from leaves of *A. articulata* significantly reduced glycemia in glucose- and alloxan-induced diabetic mice, likely due to saponin content [72]. On the other hand, *S. herbacea* exhibited multiple *in vivo* antidiabetic effects. Ethyl acetate fractions from a methanol extract from aerial organs prevented hyperglycemia in STZ-induced diabetic rats [135], while enzymatic hydrolysates from aerial organs reduced glucose and cholesterol levels [77]. Notably, dried biomass from whole plants of *S. herbacea* also decreased body weight and insulin levels in high-fat-diet-fed mice [134]. However, its use in traditional medicine remains undocumented. *Cressa* cretica demonstrated significant antidiabetic effects in *in vivo* studies using streptozotocin (STZ)-induced diabetic rats. Methanol and petroleum ether extracts led to reduced blood glucose levels, likely attributed to the phenolic and flavonoid content [90]. Hot water extracts from the leaves of *P. indica* effectively reduced hyperglycemia in a dose-dependent manner in high-fat diet-induced (HFD) diabetic mice, as demonstrated by Sirichaiwetchakoon et al. [140]. Water extracts of the leaves of *Atriplex halimus* exhibited substantial blood glucose reduction in STZ-induced diabetic rats, probably related to its content in tannins and flavonoids [73].

Some species without documented ethnomedicinal use for diabetes management exhibit significant antidiabetic properties. For instance, a water extract from the leaves of *Lycium europaeum* demonstrated significant effects in *in vivo* studies on alloxan-induced diabetic adult Wistar rats [142]. The extract increased HDL-C levels while reducing blood glucose, total cholesterol (TC), low-density lipoprotein cholesterol (LDL-C), and triglycerides (TG) [142]. These effects are likely associated with its rich content of polyphenols and flavonoids [142]. Such findings highlight the potential of *L. europaeum* as a natural source for antidiabetic therapies, warranting further exploration of its bioactive compounds and mechanisms of action. Several STPs documented in Table 1 for their ethnomedicinal applications in managing diabetes have not yet been scientifically validated through *in vivo* or *in vitro* studies, as evidenced by their absence in Table 2. These species hold significant potential for further research, as their traditional use suggests bioactive properties that may contribute to glycemic control. Examples include *Tamarix gallica*, traditionally used in Algeria, Greece, Italy, Kuwait, and Portugal [57,98,99,100,101], *Juncus maritimus* in Algeria, Germany, and Lesotho [57,91,158], or *Gomphrena globosa* in Brazil and Trinidad and Tobago [76,102].

### 4.3. Hypertension

Traditionally used in Chinese medicine, *Chrysanthemum indicum* is known for its ability to alleviate cardiovascular conditions, including hypertension. *In vivo* studies confirmed its antihypertensive activity, with linarin-enriched extracts significantly reducing blood pressure by inhibiting the renin–angiotensin system (RAS) in hypertensive rats [149]. The reduction in angiotensin II production highlights a direct mechanism of action that aligns with its traditional use.

*Artemisia scoparia* has long been used in traditional medicine for treating hypertension, particularly in Asian countries. *In vivo* studies demonstrated that hot water extracts of *A. scoparia* reduced blood pressure in spontaneously hypertensive rats [148]. The antihypertensive effects were attributed to compounds such as caffeic acid, salicylic acid, and glucopyranosides, which modulated blood pressure by reducing angiotensin-converting enzyme (ACE) activity and increasing vascular endothelial growth factor (VEGF) expression. These findings validate its ethnomedicinal use and highlight its potential as a natural antihypertensive agent.

Known for its traditional applications in Brazil and Trinidad and Tobago, *Gomphrena globosa* has also shown significant antihypertensive effects in experimental studies. Ethanol extracts reduced arterial blood pressure in male dogs without affecting heart rate, likely due to its rich content of alkaloids, saponins, and coumarins [144]. This supports its ethnomedicinal reputation for managing cardiovascular disorders, including hypertension.

*Apocynum venetum*, used in traditional Chinese medicine, exhibited high antihypertensive effects in hypertensive rat models. Water and ethanol extracts of its leaves reduced blood pressure, enhanced sodium and potassium excretion, and increased urine volume [145,146,147]. The mechanisms included endothelium-dependent vasodilation mediated by NO release, providing pharmacological support for its traditional use. Other species with *in vitro* antihypertensive effects but without documented ethnomedicinal uses include *Crithmum maritimum* and *Mesembryanthemum crystallinum*. In contrast, species with ethnomedicinal applications yet to be experimentally validated include *Artemisia campestris* L. ssp. *maritima* and *A. scoparia*.

### 4.4. Cardiovascular Disorders

Species traditionally used for cardiovascular health have demonstrated efficacy in preclinical studies. For example, *Thespesia populnea* hydroalcoholic extracts improved cardiac function in doxorubicin-induced cardiotoxicity in rats, attributed to kaempferol [94]. Most species reported to have activity against cardiovascular disorders, such as *Atriplex halimus*, *Nitraria retusa*, and *Salsola kali* (Table 1), lack *in vitro* or *in vivo* experimental validation. This gap highlights their untapped potential for further exploration and scientific confirmation. On the other hand, although there is no reported ethnomedicinal use towards cardiovascular disorders, *Salicornia ramosissima* ethanol extracts significantly reduced oxidative stress markers and infarct volume in a transient middle cerebral artery occlusion model, linked to its caffeoylquinic acid derivatives and flavonoids [151].

### 4.5. Obesity

Several species traditionally used for managing obesity have shown efficacy in preclinical studies. *Salicornia herbacea* powder supplementation reduced body weight, hepatic triglycerides, and serum leptin levels in high-fat-diet-fed mice, likely through the regulation of lipid anabolic gene expressions such as SREBP-1 and PPARγ [134]. The aqueous–ethanolic extract (50%) of the same species inhibits adipogenesis by suppressing the pro-adipogenic transcription factors in 3T3-L1 preadipocytes and prevents obesity by regulating the blood lipid profile as well as the weight of adipose tissue [159]. Similarly, Bassia scoparia ethanol extracts inhibited pancreatic lipase activity and reduced triglyceride levels in obese mice, attributed to its saponins and momordin derivatives [117].

Non-traditional species also show promise in obesity management. For example, *Limonium tetragonum* ethyl acetate fractions decreased adipocyte differentiation *in vitro* by suppressing adipogenesis-related transcription factors such as PPARγ and SREBP-1, and reducing lipid accumulation [156]. Additionally, *Pluchea indica* extracts inhibited adipogenesis in 3T3-L1 cells and improved dyslipidemia and obesity markers in high-fat-diet-fed mice [140]. These findings suggest the potential of non-traditional STPs in addressing obesity-related metabolic disorders. Other species with ethnomedicinal uses towards obesity still lack validation, including, for example, *Tamarix gallica* and *Armeria maritima*.

## 5. Promising Bioactive Molecules from STPs for Managing MetS

**Figure 3:**
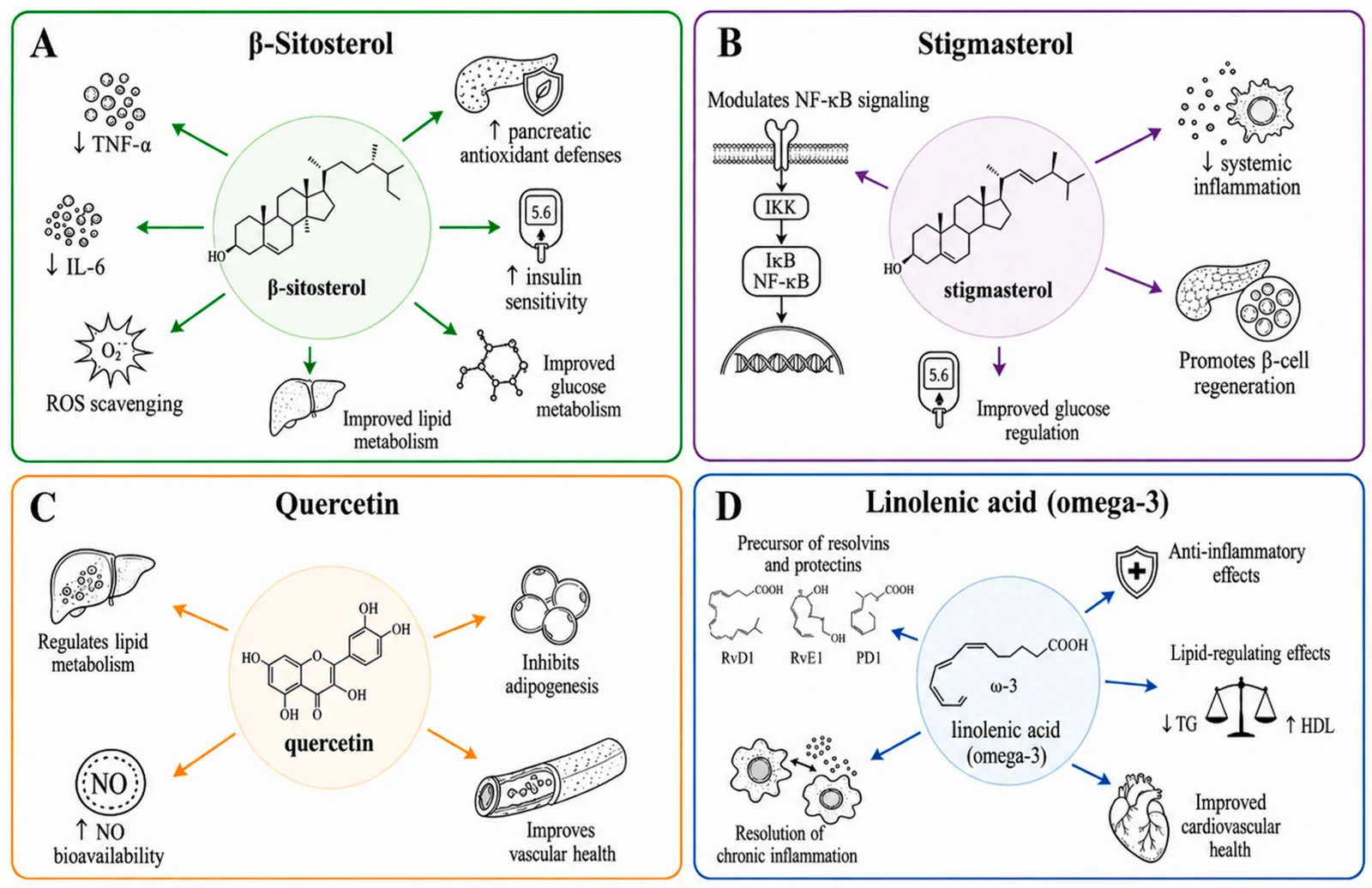
Schematic representation of the main mechanisms of action of selected bioactive compounds relevant to metabolic syndrome. (**A**) β-sitosterol, showing anti-inflammatory, antioxidant, and metabolic effects, including reduction in TNF-α and IL-6, reactive oxygen species (ROS) scavenging, enhancement of pancreatic antioxidant defenses, and improvement of insulin sensitivity, glucose metabolism, and lipid metabolism. (**B**) Stigmasterol, illustrating modulation of NF-κB signaling, reduction in systemic inflammation, promotion of β-cell regeneration, and improvement of glucose regulation. (**C**) Quercetin, highlighting regulation of lipid metabolism, inhibition of adipogenesis, increased NO bioavailability, and improvement of vascular health. (**D**) Linolenic acid (omega-3), showing its anti-inflammatory and lipid-regulating effects, and improvement of cardiovascular health. Figure created with the assistance of an AI tool and subsequently reviewed and edited by the authors.

Halophytes and other STPs represent a rich source of bioactive compounds with significant therapeutic potential for addressing MetS and its associated disorders. This section discusses the most promising bioactive compounds, selected from the previous sections, their roles in metabolic regulation, and their potential for further research and therapeutic development. Their main mechanisms of action, relevant to MetS, are schematically illustrated in Figure 3.

### 5.1. Inflammation

Isorhamnetin-3-*O*-β-D-glucopyranoside, a glycosylated flavonoid isolated from *S. herbacea,* has shown relevant anti-inflammatory properties. It inhibits the activation of hepatic stellate cells through modulation of transforming growth factor-β, reducing the production of inflammatory mediators [126]. Similar benefits have been attributed to terpenoids such as α-pinene, linalool, and linalyl acetate from *Chenopodium album*, which target key inflammatory pathways by suppressing COX-2 expression and oxidative stress [160]. β-Sitosterol and stigmasterol, two phytosterols identified in *P. maritimum*, exhibit complementary anti-inflammatory effects by inhibiting NF-κB signaling, scavenging reactive oxygen species, and modulating pro-inflammatory cytokines [161,162,163]. Additionally, linolenic acid, an omega-3 fatty acid, serves as a precursor for resolvins and protectins, compounds that actively resolve inflammation while improving cardiovascular health [164]. Compounds like 5-hydroxymethyl-2-furfural and (S)-asfural, derivatives found in *Asparagus officinalis*, further contribute to inflammation management by reducing oxidative stress and cytokine production, highlighting their potential for chronic inflammatory conditions [129,165].

### 5.2. Diabetes

Recent studies have explored the potential antidiabetic effects of β-sitosterol, stigmasterol, 1-octacosanol, and linolenic acid, identified in *P. maritimum*, highlighting their roles in modulating glucose metabolism and improving insulin sensitivity. For example, β-Sitosterol improves glucose metabolism by lowering glycated hemoglobin and enhancing pancreatic antioxidant levels [166], while stigmasterol aids in GLUT4 translocation, β-cell regeneration, and reduced fasting glucose levels [162]. Isorhamnetin-3-*O*-β-D-glucoside, a flavonoid glycoside isolated from *S. herbacea*, inhibits α-glucosidase, thus reducing postprandial glucose levels, and mitigates diabetic complications such as neuropathy by inhibiting aldose reductase activity [136,167].

Acteoside, a phenylethanoid glycoside from *C. phelypaea*, improves insulin sensitivity and lipid profiles in diabetic models [168], while echinacoside, another phenylethanoid glycoside, has been reported to possess antidiabetic activity [169] and is capable of alleviating T2DM-induced injury in the db/db mouse model and insulin-resistant LO2 cell model by mediating the AKT pathway [170]. Catechin, a flavonoid predominantly found in green tea, enhances glucose uptake and reduces oxidative stress linked to insulin resistance [171].

### 5.3. Hypertension

Linarin, a glycosylated flavonoid from *D. indicum*, demonstrates effective blood pressure reduction by inhibiting the renin–angiotensin system without adverse effects on kidney or liver function [149]. Eugenol derivatives identified in *Artemisia scoparia* exhibit vasodilatory activity by modulating endothelial NO pathways and adrenergic receptors [172,173]. Similarly, caffeic acid and quercetin derivatives have been shown to enhance endothelial function, reduce vascular resistance, and improve NO bioavailability, with clinical studies confirming their effectiveness in managing blood pressure [174]. These bioactive compounds offer a natural and multifaceted approach to hypertension treatment.

Caffeoylquinic acid derivatives, flavonoids, and fatty acids from *S. ramosissima* show significant potential in managing cardiovascular disorders due to their antioxidant, anti-inflammatory, and lipid-regulating properties. Caffeoylquinic acids reduce oxidative stress by scavenging ROS and inhibiting lipid peroxidation, protecting vascular tissues and preventing LDL oxidation, a critical step in atherosclerosis progression [175,176]. Their lipid-lowering effects further enhance their role in regulating cholesterol levels and mitigating cardiovascular risk [177].

Kaempferol, a flavonoid from *T. populnea*, has demonstrated antioxidant and anti-inflammatory activities, protecting endothelial cells from oxidative damage and reducing the expression of pro-inflammatory cytokines like TNF-α and IL-6 [178]. Additionally, it improves lipid profiles by lowering LDL and increasing HDL cholesterol, while inhibiting LDL oxidation, which prevents plaque formation. Its vasodilatory effects, mediated by enhanced NO bioavailability, further contribute to improved endothelial function and reduced blood pressure, making it a promising candidate for addressing hypertension and other cardiovascular risk factors [179].

### 5.4. Obesity

Caffeoylquinic acid derivatives from *S. ramosissima* and *P. indica* inhibit adipogenesis by suppressing transcription factors like PPARγ and enhancing antioxidant defenses, which improve insulin sensitivity and reduce fat accumulation [180,181]. Beta-caryophyllene, isolated from *P. indica*, acting as a CB2 receptor agonist, alleviates inflammation in adipose tissue and supports lipid metabolism [182]. Quercetin and its derivatives, isolated from, for example, *C. maritimum*, target oxidative stress, inflammation, and lipid metabolism, addressing multiple obesity-related complications [183,184,185]. From *S. herbacea* derivatives, like isorhamnetin-3-*O*-glucoside, further modulate lipid metabolism by inhibiting adipocyte differentiation and promoting lipid catabolism, making them valuable in weight management strategies [159,186,187].

## 6. Bioactive Molecules with Multiple Effects

Several bioactive molecules derived from halophytes and other STPs exhibit multiple bioactivities, making them particularly promising for managing MetS (Figure 2). Among these, β-sitosterol and stigmasterol stand out for their ability to simultaneously modulate inflammatory and oxidative stress pathways while improving lipid metabolism and glucose homeostasis. β-Sitosterol inhibits pro-inflammatory cytokines like TNF-α and IL-6, reduces oxidative stress by scavenging ROS, and enhances pancreatic antioxidant defenses, which collectively improve insulin sensitivity and glucose metabolism [161,163]. Stigmasterol complements these effects by modulating NF-κB signaling and promoting β-cell regeneration, further supporting its role in glucose regulation and systemic inflammation reduction [162]. Quercetin also demonstrates multifaceted benefits, including lipid metabolism regulation, inhibition of adipogenesis, and vascular health improvement through enhanced NO bioavailability [183,184,185]. Additionally, linolenic acid, an omega-3 fatty acid, provides anti-inflammatory and lipid-regulating effects, serving as a precursor for resolvins and protectins, which actively resolve chronic inflammation while improving cardiovascular health [164,188]. These compounds, with their overlapping mechanisms targeting multiple metabolic pathways, offer a synergistic approach to managing the complex pathophysiology of metabolic syndrome, highlighting their potential for integration into nutraceuticals or functional foods aimed at comprehensive metabolic health improvement.

## 7. Comparing Bioactive Molecules from STPs with Clinically Relevant Synthetic Ligands

The pharmacological profile of the four STP compounds highlighted above is best understood through direct mechanistic comparison with the most clinically relevant synthetic ligands. In this regard. and rather than matching each STP compound to a single comparator, the discussion below organizes the comparison around the principal mechanistic axes that drive MetS pathophysiology: (i) NF-κB/JNK-driven inflammation, (ii) PPARγ-mediated insulin sensitization, (iii) PPARα-driven β-oxidation and dyslipidemia, and (iv) the resolution phase of inflammation. For each axis, the synthetic ligand(s) selected are those with an established clinical track record and an explicitly characterized molecular mechanism that allows a meaningful head-to-head comparison: metformin (the gold-standard first-line antidiabetic), pioglitazone and rosiglitazone (the prototype full PPARγ agonists), fenofibrate (the prototype PPARα agonist in current clinical use), and aspirin/conventional NSAIDs (the prototype approach to blocking pro-inflammatory lipid mediators) [189,190,191,192]. This selection was guided by the principle that a reference ligand must share a defined molecular axis with the natural product but differ in the breadth of its polypharmacology, so that both convergences and divergences can be clearly identified.

### 7.1. β-Sitosterol Versus Metformin and Thiazolidinediones

β-Sitosterol converges on the same IKKβ/NF-κB/JNK axis targeted by metformin, but through a distinct upstream logic. Metformin acts primarily by inhibiting mitochondrial complex I, which suppresses hepatic cAMP signaling and reduces gluconeogenesis [190]. In a high-fat diet/sucrose-induced T2DM rat model, β-sitosterol at 20 mg/kg simultaneously downregulated IKKβ/NF-κB and JNK gene and protein expression in adipose tissue, restored insulin receptor and GLUT4 expression, normalized adiponectin, and suppressed SREBP-1c. The study explicitly reported that β-sitosterol acted as effectively as metformin in circumventing inflammation and insulin resistance [193,194].

Relative to the thiazolidinediones pioglitazone and rosiglitazone, β-sitosterol behaves as a selective PPARγ modulator. While TZDs are full PPARγ agonists that drive mesenchymal stem cell differentiation into adipocytes, increase adiponectin, and decrease hepatic and peripheral triglycerides, β-sitosterol upregulates PPARγ expression while coupling this to anti-inflammatory cytokine regulation rather than full adipogenic induction [195,196]. This pattern coincides with the broader characterization of natural PPARγ ligands (honokiol, amorfrutin 1, amorphastilbol) that occupy the PPARγ ligand-binding domain in a binding mode distinct from full glitazone agonists and improve metabolic parameters in diabetic animal models with reduced side effects [196].

### 7.2. Stigmasterol Versus Pioglitazone and Rosiglitazone

Stigmasterol recapitulates the insulin-sensitizing profile of rosiglitazone in 3T3-L1 adipogenic differentiation assays, showing dose-dependent insulin-like and insulin-sensitizing activity comparable to the thiazolidinedione positive control, albeit with reduced stimulus in the absence of insulin [197]. Independently, stigmasterol has been shown to increase glucose uptake via GLUT4 translocation through an AMPK/PPAR-related mechanism, the same cellular entry point engaged by TZDs [195,196]. The key divergence lies in the inflammatory layer. TZDs act essentially as full PPARγ agonists whose clinical utility is limited by cardiovascular, weight-gain, and bone-related adverse effects [190]. Stigmasterol, by contrast, simultaneously inhibits the NF-κB/p65 subunit, a property documented across multiple phytosterols (β-sitosterol, ergosterol, stigmasterol), and suppresses interconnected MAPK cascades such as ERK and p38 [198,199]. The molecular-docking characterization of natural neolignans (dieugenol, tetrahydrodieugenol, magnolol) is instructive: these compounds bound the PPARγ LBD with nanomolar EC_50_ values in a manner highly similar to pioglitazone, yet behaved as partial agonists in cell-based reporter assays and showed reduced adipogenic side effects [200]. Stigmasterol appears to follow a similar functional profile, supporting the notion that phytosterols may act as selective PPAR modulators (SPPARMs) rather than full PPAR agonists [196].

### 7.3. Quercetin Versus Fibrates and Synthetic PPARγ Agonists

Quercetin is a weak partial agonist of PPARγ that improves insulin-stimulated glucose uptake in mature 3T3-L1 adipocytes without promoting the differentiation of preadipocytes [201]. Molecular docking in 3T3-L1 cells demonstrated a significant correlation between binding affinity to the PPARγ LBD and anti-adipogenic activity in the early differentiation phase, with the residues Phe264, His266, Ile281, Cys285 and Met348 being the most frequently involved in ligand interaction. These residues differ from those predominantly engaged by full glitazone agonists, which explains why quercetin inhibits adipogenesis by downregulating *Scd1* and *Lpl* without inducing the lipid accumulation that typifies TZD treatment [202].

In contrast to TZDs, which act almost exclusively through PPARγ, quercetin simultaneously modulates PPARγ, C/EBPα, SREBP-1c, FAS, ATGL, HSL, LPL and aP2 in OP9 stromal cells, a multi-target engagement that no single glitazone achieves [203]. In obese Zucker rats, chronic quercetin treatment at 10 mg/kg for 10 weeks reduced systolic blood pressure, plasma triglycerides, total cholesterol, free fatty acids, insulin and visceral adipose tissue TNF-α, while upregulating eNOS and downregulating iNOS—a nitric-oxide bioavailability restoration not produced by TZDs or fibrates [204]. Quercetin also counteracted hypoxia-induced dysregulation of adipokines (Angptl4, adipsin, irisin, PAI-1) and glycolytic genes (ENO2, PFKP, PFKFB4) in SGBS human adipocytes, an effect linked to both free-radical scavenging and direct mitochondrial modulation [205].

Against fibrates (fenofibrate, bezafibrate), which are synthetic PPARα agonists that reduce triglycerides via upregulation of fatty acid β-oxidation and downregulation of apolipoprotein C-III, quercetin exhibits much weaker and mixed PPARα/γ agonism that does not match the triglyceride-lowering potency of fibrates but offers complementary cardiovascular and adipogenic benefits [189]. A direct head-to-head comparison in NAFLD patients with hypertriglyceridaemia showed that omega-3 carboxylic acids shifted the circulating mediator profile towards CYP-/LOX-derived lipid mediators (17-HDHA, 18-HEPE, 19,20-DiHDPA) from EPA and DHA, while fenofibrate preferentially reduced pro-atherogenic ceramides; both strategies ameliorated chronic inflammation, but through entirely non-overlapping lipidome reprogramming [206], a finding consistent with the view that combining a PPARα-driven fibrate-like axis with the multi-target profile of an STP-derived compound may produce additive benefits [189,206].

### 7.4. Linolenic Acid Versus Aspirin, NSAIDs and Corticosteroids

Linolenic acid’s mechanism is mechanistically orthogonal to that of NSAIDs and corticosteroids. NSAIDs block COX-1/COX-2 and corticosteroids broadly repress pro-inflammatory gene transcription, both approaches being heavily suppressive and leaving resolution biology unaddressed [191,207]. ALA is enzymatically elongated to EPA and DHA, which are converted within the resolution phase of acute inflammation into specialized pro-resolving mediators, the resolvin and protectin families, that actively signal the termination of inflammation through specific receptors and produce anti-inflammatory, pro-resolving and anti-fibrotic phenotypes [191,207,208].

The relationship to aspirin is particularly revealing. Aspirin “jump-starts” resolution by acetylating COX-2 and triggering biosynthesis of “aspirin-triggered” epimers of resolvins and lipoxins but cannot generate the full SPM cascade in the absence of substrate [191]. ALA provides exactly that endogenous substrate. As shown in a randomized placebo-controlled crossover study in NAFLD patients, omega-3 carboxylic acids reduced pro-inflammatory PGE2, PGE1, PGD1 and thromboxane B2 while increasing CYP-/LOX-derived lipid mediators, confirming that ALA-derived substrates engage resolution pathways that aspirin (without substrate) and conventional NSAIDs (without induction of resolution) cannot [206]. This makes ALA’s mechanism fundamentally complementary rather than redundant with synthetic anti-inflammatory drugs.

Across these comparisons, a coherent pattern emerges. Regarding similarities, all four STP compounds act on canonical MetS-related axes (NF-κB/JNK, PPARγ, PPARα, SPM resolution) that are also engaged by their respective synthetic counterparts; dose–response effects have been demonstrated *in vivo* at pharmacologically relevant concentrations [193,194,196,204]. As for differences, the STP compounds consistently behave as partial modulators of nuclear receptors rather than full agonists [197,201,203], integrate antioxidant capacity as a structural feature of the flavonoid/sterol scaffold rather than as a separate drug component [193,194,204], and act on multiple axes simultaneously rather than along a single signaling pathway.

Three integrated advantages emerge from this comparative analysis. First, polypharmacology with reduced liability: these compounds deliver full-agonist-comparable metabolic improvements while avoiding the canonical side effects of TZDs (weight gain, fluid retention, bone loss) [190,196,200]. Second, simultaneously addressing inflammation resolution and oxidative stress: the integration of antioxidant and pro-resolving properties within the same molecular scaffold is rarely achieved by a synthetic ligand. Third, integration into functional-food and nutraceutical matrices: because these compounds occur naturally in edible STP sources and food-grade omega-3 oils, they lend themselves to dietary strategies that combine several compounds in a single formulation, thereby targeting multiple MetS pathways with reduced pharmacological burden and improved long-term adherence [189,190,191,206].

Integrating these observations, the STP-derived compounds can be regarded as multi-target modulators that combine SPPARM-like receptor behavior with intrinsic antioxidant capacity and, in the case of ALA, substrate-driven engagement of the resolution phase of inflammation. This pharmacological breadth positions them as particularly attractive candidates for nutraceutical and functional-food strategies aimed at the multifactorial pathophysiology of MetS.

## 8. Pharmacokinetic Limitations of STP-Derived Compounds and the Promise of Next-Generation Delivery Systems

While Section 6 has documented the *in vitro* and *in vivo* activities of STP-derived compounds and Section 7 has situated them relative to clinically established synthetic ligands, translation of these benefits to human therapy is constrained by pharmacokinetic limitations that remain largely unaddressed in the current MetS nutraceutical landscape [195,209]. This subsection examines the main bioavailability bottlenecks of each compound category and the emerging formulation strategies that may overcome them.

### 8.1. Phytosterols (β-Sitosterol, Stigmasterol): Lipophilicity as the Governing Constraint

The phytosterols β-sitosterol and stigmasterol are highly lipophilic and poorly soluble in water, features that strongly limit their intestinal absorption [195]. Their low bioavailability is consistent with the lipophilicity-limited absorption pathway of C29 sterols, characterized by poor membrane penetration, meal-dependent uptake and extensive biliary excretion [209,210]. As a result, phytosterol-enriched functional foods, such as margarines, yogurts and beverages, generally require gram-range daily doses to achieve clinically relevant LDL-C reductions, which may limit their applicability for broader MetS-related outcomes, including adipose-tissue inflammation and ectopic fat deposition [209,210].

To overcome these limitations, several delivery strategies have been explored. For example, nanostructured lipid carriers (NLCs) have improved β-sitosterol solubility, digestive stability and bioaccessibility, while also producing stronger reductions in plasma total cholesterol and LDL-C in mice than non-formulated β-sitosterol suspensions [211]. Similarly, chitosan/tripolyphosphate nanoparticles loaded with stigmasterol-rich *Artemisia lactiflora* extracts showed high entrapment efficiency, nanoscale particle size and enhanced *in vitro* release under simulated gastric conditions [212]. More broadly, colloidal systems such as emulsions, oleogels, liposomes and polymeric nanoparticles have been shown to improve phytosterol water-dispersibility, stability and bioavailability, supporting their incorporation into a wider range of cholesterol-lowering functional foods and beverages [210].

Overall, lipid- and polymer-based nanocarriers represent promising strategies to improve phytosterol intestinal solubilization, absorption reproducibility and sustained release. These advances may help translate the NF-κB/JNK- and PPARγ-modulating activities discussed in Section 7 into clinically relevant MetS-related outcomes at lower daily doses [209,210,211].

### 8.2. Flavonoids: Solubility, First-Pass Metabolism and Microbiota Variability

Quercetin has established pharmacodynamic activity but limited pharmacokinetic feasibility; therefore, its application in the pharmaceutical field is limited by poor aqueous solubility, low oral bioavailability, poor permeability and instability under gastrointestinal conditions, which collectively prevent systemic concentrations reached *in vitro* from being replicated *in vivo* [213,214]. This limitation is compounded by phase II metabolism: quercetin undergoes extensive glucuronidation, sulfation and methylation in the gut, transforming the aglycone into more hydrophilic but often less biologically active metabolites, and its absorption occurs predominantly in the small intestine after deglycosylation by the gut microbiota [215,216]. For quercetin glycosides such as myricitrin, the prerequisite hydrolysis step introduces additional inter-individual variability driven by microbiota composition, contributing to the heterogeneous clinical responses observed across supplementation trials [215,216].

Different approaches have been studied to surpass these limitations. For example, self-nanoemulsifying drug delivery systems containing quercetin (oil, surfactant and co-surfactant) protect the flavonoid from gastric degradation and substantially improve oral bioavailability. In an *in vivo* model of metabolic syndrome, a quercetin-loaded SNEDDS restored the vascular reactivity compromised by MetS and advanced glycation end-products, providing the first evidence that the eNOS/iNOS-modulating activity discussed in Section 7 can be maintained when quercetin is delivered through an enabling formulation [213]. On the other hand, lipid-based nanocarriers, such as liposomes, nanostructured lipid carriers, and solid-lipid nanoparticles, are widely used to improve oral absorption, target antioxidant activity to specific tissues, and ensure sustained release of quercetin [216].

### 8.3. Linolenic Acid and Polyunsaturated Fatty Acids: Oxidative Vulnerability and Enteric Stability

α-Linolenic acid, while an essential fatty acid, suffers from low intestinal bioavailability and a high oxidative potential that limit its nutritional and pharmacological performance. As an ω-3 PUFA, ALA is prone to oxidative deterioration when exposed to oxygen, heat, and moisture, generating undesirable flavors and reducing nutritional value before the compound can reach its enzymatic conversion to EPA, DHA, and downstream specialized pro-resolving mediators [217]. Nanoemulsions prepared from food-grade ingredients are the most investigated system for ω-3 PUFA delivery, providing stability against oxidation, masking off-flavors and increasing intestinal bioavailability; nanoemulsion formulations have also been successfully used to enhance the bioaccessibility of curcumin and EGCG, highlighting their versatility for combined polyphenol + PUFA matrices [218]. Liposomes, microencapsulation and solid lipid nanoparticles/nanostructured lipid carriers have been reviewed as emerging oral delivery systems for PUFAs that improve oxidative stability across storage, protect PUFAs during gastrointestinal transit and increase intestinal absorption [217]. Encapsulation strategies (physical blending, interesterification and encapsulation proper, with the latter being most effective for oxidatively sensitive PUFAs) have been compared for their impact on omega-3 bioavailability, with the general conclusion that the encapsulation technique selected must be tailored to the target food matrix and the desired release profile [219].

### 8.4. Translational Implications for MeTs

When the bioavailability bottlenecks of each compound class are considered together, a coherent translational framework emerges. All four categories of STP-derived compounds highlighted in this review suffer from absorption or stability limitations that are not encountered, or are managed differently, by the synthetic reference ligands discussed in Section 7. Metformin, pioglitazone and fenofibrate were engineered as oral immediate-release formulations with predictable absorption windows; the STP bioactives, by contrast, are predominantly GRAS food constituents whose delivery format can be reformulated to bypass their inherent pharmacokinetic weaknesses [195,220,221].

These findings have three main integrative implications. First, lipid-based nanosystems may reduce the effective dose required to achieve bioactivity. NLC and SMEDDS formulations of β-sitosterol and stigmasterol, as well as nanoemulsion-based α-linolenic acid carriers, support the use of sub-200 nm lipid carriers to enhance bioavailability and biological efficacy at lower doses than the free compounds. This is particularly relevant for long-term nutraceutical or formulation-based strategies targeting the multifactorial pathophysiology of MetS, where safety, tolerability and drug–nutrient interaction risks are strongly influenced by dose [211].

Second, these delivery systems may facilitate combination therapy within a single matrix. Lipid-based nanocarriers can co-load lipophilic phytosterols and polyunsaturated fatty acids, while hydrogels based on chitosan or alginate can incorporate compounds such as quercetin and EGCG. This supports a multi-target strategy involving parallel modulation of IKKβ/NF-κB/JNK, PPARγ/PPARα, eNOS/iNOS and the SPM cascade, consistent with the polypharmacological potential attributed to STP-derived compounds [216,219,222].

Third, formulation-enabled delivery may provide a more realistic pathway for clinical translation. By combining the chronic oral safety profile of food-grade bioactives with improved pharmacokinetic performance, these systems may help bridge the gap between robust *in vitro* or animal evidence and the inconsistent outcomes often reported in human nutraceutical trials for MetS, which are frequently limited by poor formulation, low bioavailability, and weak pharmacokinetic–pharmacodynamic predictability [221].

## 9. Conclusions, Remarks, and Future Perspectives

This review highlights the potential of halophytes and other STPs as sources of bioactive compounds with relevance for the management of MetS-related conditions, including inflammation, diabetes, cardiovascular disorders, dyslipidemia and obesity. Species such as *C. album*, *L. spathulatum*, *D. indicum*, *M. crystallinum*, *S. herbacea* and *L. tetragonum* have shown relevant *in vitro* and/or *in vivo* bioactivities associated with these conditions and should be further investigated. In addition, several species with ethnomedicinal uses remain poorly explored from a pharmacological and mechanistic perspective. Among them, *I. pes-caprae*, *Z. coccineum*, *T. gallica*, *S. fruticosa*, *A. maritima*, *A. littoralis*, *S. vera*, *L. maritima*, *B. gymnorhiza* and *R. mangle* emerge as particularly interesting candidates, as they combine traditional uses related to inflammation, diabetes, cardiovascular disorders or obesity with limited direct experimental validation. These findings underscore the still underexplored potential of STPs as sources of bioactive compounds and support the need for further phytochemical characterization, bioactivity-guided fractionation, mechanistic studies and preclinical validation.

Among the most relevant metabolites, β-sitosterol and stigmasterol stand out for their ability to modulate inflammation, oxidative stress, lipid metabolism and glucose homeostasis. β-Sitosterol has been associated with reduced pro-inflammatory cytokines, ROS scavenging, improved antioxidant defenses and enhanced insulin sensitivity, whereas stigmasterol may contribute to NF-κB inhibition, β-cell protection or regeneration, and improved glucose regulation. Quercetin further supports metabolic and cardiovascular protection by targeting inflammation, oxidative stress, adipogenesis and NO bioavailability. Linolenic acid, as an omega-3 fatty acid precursor of specialized pro-resolving mediators such as resolvins and protectins, may contribute to the resolution of chronic inflammation, lipid regulation and improved insulin sensitivity. Together, these compounds act on interconnected features of MetS, including chronic inflammation, insulin resistance, dyslipidemia and vascular dysfunction.

Despite this promise, important translational and safety-related challenges remain. The potential use of halophytes and other STPs as sources of bioactives for MetS management should consider, for example, the need for long-term safety data for chronic intake of nanoformulated phytosterols, flavonoids and polyunsaturated fatty acids; better understanding of pharmacokinetic variability under fed and fasted conditions and its interaction with gut microbiota-mediated metabolism; scalable food-grade manufacturing processes for NLC, SMEDDS and related delivery systems; and well-designed clinical trials comparing formulated STP-derived bioactives with reference compounds or standard interventions.

Finally, future studies should move beyond biomarker changes and include clinically relevant MetS endpoints, such as T2DM incidence and cardiovascular outcomes. Addressing these gaps will be essential to translate the ethnomedicinal, mechanistic and preclinical evidence summarized in this review into evidence-based nutraceutical, functional-food or therapeutic strategies for MetS management.

## Figures and Tables

**Table 2:** Bioactive properties of halophytes and other salt-tolerant plants, relevant for the management of metabolic syndrome (MetS) related conditions, namely inflammation, diabetes, hypertension, cardiovascular disorders, and obesity.

Family	Species	Plant Organ	Assay	Extract/Compound	Chemical Composition of the Extract or Bioactive Compounds Isolated/Identified	Assay	Results	References
Inflammation								
Aizoaceae	*Carpobrotus edulis* (L.) N.E.Br.	Leaves	*In vitro*	50% methanol extract, 70% acetone and water extract	High total phenolic and flavonoid contents	α-15-LOX inhibition	Acetone extract: IC_50_ = 22.3 μg/mL; water extract: IC_50_ = 59.8 μg/mL	[123]
Amaranthaceae	*Chenopodium album* L.	Leaves	*In vivo*	Essential oil	α pinene, linalool and linalyl acetate	12-*O*-tetradecanoylphorbol-13-acetate (TPA)—induced ear edema in mice	Reduction in ear edema	[124]
		Aerial parts	*In vitro*	Dichloromethane fraction of methanol extract	Loliolide, methylethylmalemide and benzoic acid	Inhibition of the lipopolysaccharide (LPS)-induced production of nitric oxide (NO) in the murine macrophage RAW 264.7 cell line	IC_50_ = 81.7 μg/mL	[125]
	*Salicornia europaea* L. (=*Salicornia herbacea* L.)	Aerial organs	*In vitro*	Isorhamnetin 3-*O*-beta-d-glucopyranoside isolated from a crude CH(2)Cl(2)/methanol extract	n.a.	LPS—stimulated RAW 264.7 macrophages	Suppression of LPS induced NO production; expression of the cytokines inducible NO synthase, tumor necrosis factor-alpha, and interleukin-1beta	[126]
	*Suaeda fruticosa* Forssk. ex J.F.Gmel.	Shoots	*In vitro*	Methanol extract	n.a.	LPS—stimulated RAW 264.7 macrophages	66.4% of NO reduction at 160 µg/mL	[127]
Asparagaceae	*Asparagus officinalis* L.	Leaves	*In vitro*	Linoleic acid isolated from an ethyl acetate-soluble fraction of the methanol extract	n.a.	Cyclooxygenase-1 (COX-1) and COX-2 inhibition assay	COX-1: IC_50_ = 14.6 ug/mL; COX-2, IC_50_ = 0.53 mg/mL	[128]
	*A. officinalis*	Stems	*In vitro*	Enzyme-treated asparagus extract (ETAS™)	5-hydroxymethyl-2-furfural and (S)-asfural	Primary cultured rat hepatocytes treated with IL-1β	Block of the release of NO, inhibition of the production of pro-inflammatory cytokines and chemokines.	[129]
Chenopodiaceae	*Atriplex portulacoides* L. (=*Halimione portulacoides* (L.) Aellen)	Leaves	*In vitro*	Chloroform extract	Moderate phenolics content	LPS—stimulated RAW 264.7 macrophages	IC_50_ = 109 μg/mL	[130]
Malvaceae	*Thespesia populnea* (L.) Sol. ex Corrêa	Leaves	*In vivo*	Methanol extract	n.a.	Carrageenan and formaldehyde-induced paw edema of BALB/c male mice	Pronounced reduction in the volume of paw edema	[130]
Plantaginaceae	*Plantago coronopus* L.	Leaves	*In vitro*	Hexane extract	Low phenolics content	LPS—stimulated RAW 264.7 macrophages	IC_50_ = 98 μg/mL	[131]
Plumbaginaceae	*Limonium spathulatum* (Desf.) Kuntze	Aerial parts	*In vitro*	Chloroform, methanol, and methanol:water (5:1) extracts	High levels of total phenolics and flavonoids	LPS—stimulated RAW 264.7 macrophages	Methanol extract: inhibition on NO releases by 86.2% at 100 µg/mL	[132]
Polygonaceae	*Polygonum maritimum* L.	Leaves	*In vitro*	Dichloromethane extract	β-sitosterol, stigmasterol, 1-octacosanol and linolenic acid.	LPS—stimulated RAW 264.7 macrophages	IC_50_ = 48 µg/mL	[130]
Poaceae	*Cynodon dactylon* (L.) Pers.	Whole plant	*In vivo*	Water extract	Glycosides and flavonoids	Carrageenan, serotonin, histamine and dextran induced rat paw edema and cotton pellet method.	Inhibition in dry-weight cotton pellet formation	[133]
Diabetes								
Aizoaceae	*C. edulis*	Leaves	*In vitro*	50% methanol, 70% acetone and water extracts	High total phenolic and flavonoid contents	α-glucosidase inhibition activity	Water extract: IC_50_ = 5 μg/mL	[123]
Amaranthaceae	*Atriplex halimus* L.	Leaves	*In vivo*	Water extract	Tannins and flavonoids	STZ diabetic induced male Wistar albino rats	Significant reduction in blood glucose level	[73]
	*Anabasis articulata* (Forssk.) Moq.	Leaves	*In vivo*	Decoction	Saponins	Glucose induced antihyperglycemic swiss albino mice and alloxan—induced diabetic swiss albino mice	Decreased glycaemia and blood glucose	[72]
	*Salicornia europaea* L. (=*Salicornia herbacea* L.)	Aerial organs	*In vivo*	Enzymatic hydrolysate	n.a.	STZ—induced diabetic rats	Decreased body-weight gain, blood glucose and cholesterol levels	[77]
		Whole plant	*In vivo*	Dried powder	n.a.	Male ICR mice fed with high-fat diet	Decreased body weight gain, liver weight, hepatic triglyceride, serum leptin and insulin; decreased mRNA level of key lipid anabolic genes	[134]
		Aerial organs	*In vivo*	50% ethanol extract	n.a.	Hyperglycemia induced by high fat diet in ICR mice	Prevention of hyperglycemia possibly by its effects on PEPCK, glucose 6-phosphatase gene expressions in liver	[135]
		Aerial organs	*In vivo*	Ethyl acetate fraction from a methanol extract	Isorhamnetin-3-*O*-beta-D-glucoside	STZ—induced diabetic rat	Ethyl acetate fraction: RLAR inhibition (IC_50_ = 0.75 µg/mL), reduced serum glucose, and decreased sorbitol accumulation in lenses, RBCs, and sciatic nerves. Isorhamnetin-3-*O*-β-D-glucoside inhibited RLAR with IC_50_ = 1.4 µM	[136]
	*S. maritima*	Whole plant	*In vitro*	Acetone extract	n.a.	α-amylase and α-glucosidase inhibition	IC_50_ = 32.6 μg/mL	[137]
Apiaceae	*Eryngium maritimum* L.,	Roots, stems, leaves and flowers	*In vitro*	Infusion, decoction, and tincture.	n.a.	Inhibition of microbial and mammalian α-glucosidase and α-amylase enzymes	Tinctures from stems, leaves and flowers inhibited the three enzymes	[138]
Convolvulaceae	*Cressa cretica* L.	Leaves	*In vivo*	Petroleum ether and methanol extracts	Phenolic compounds and flavonoids	STZ—induced Wistar diabetic rats	Reduction in blood glucose level	[90]
Orobanchaceae	*Cistanche phelypaea* (L.) Cout.	Roots, stems and flowers	*In vitro*	Water extract	Stems: acteoside; roots: echinacoside and tubuloside A.	Inhibition of α-amylase and α-glucosidase	α-amylase: stems, IC_50_ = 0.12 mmol acarbose equivalents (ACAE)/g); α-glucosidase: roots, IC_50_ = 7.05 mmol ACAE/g	[139]
Plumbaginaceae	*Armeria pungens* (*Brot.*) *Hoffmanns.* & *Link*	Flowers and leaves	*In vitro*	Ethanol and water extracts	Phenolic compounds (catechin)	Inhibition of α- glucosidase	Ethanol extract from flowers: IC_50_ = 26.7 μg/mL, water extract from leaves: IC_50_ = 75.4 μg/mL.	[66]
	*Limonium spathulatum* (Desf.) Kuntze	Aerial parts	*In vitro*	Chloroform, methanol, and methanol:water (5:1) extracts	Phenolics and flavonoids	Inhibition of α-glucosidase	Methanol extract: IC_50_ = 2.83 µg/mL; methanol:water extract: IC_50_ = 5.03 µg/mL	[132]
	*Pluchea indica* (L.) Less.	Leaves	*In vivo*	Hot water extract	n.a.	Oral glucose tolerance test (OGTT) on high fat diet-induced (HFD) mice	Reduction in hyperglycemia in a dose-dependent manner	[140]
Polygonaceae	*P. maritimum*	Roots and leaves	*In vitro*	Methanol and dichloromethane extracts	Sitosterol, stigmasterol, 1-octacosanol and linolenic acid	Inhibition of α-amylase and baker’s yeast and rat’s α-glucosidases.	Baker’s yeast a-glucosidase: methanol root extract, IC_50_ = 19 µg/mL methanol leaf extract, IC_50_ = 29 µg/mL; rat’s a-glucosidase: dichloromethane leaf extract, IC_50_ = 2527 µ/mL	[141]
*Solanaceae*	*Lycium europaeum* L.	Leaves	*In vivo*	Water extract	Polyphenols and flavonoids	Alloxan-induced diabetic adult Wistar rats	Increased high-density lipoprotein cholesterol (HDL-C) and reduced blood glucose, total cholesterol (TC), low-density lipoprotein cholesterol (LDL-C) and TG	[142]
Hypertension								
Aizoaceae	*Mesembryanthemum crystallinum* L.	Leaves and stems	*In vitro*	Purified polyphenolic extract	n.a.	Angiotensin Converting Enzyme (ACE-I) inhibition	90.5% inhibition at 1 mg/mL	[143]
Amaranthaceae	*Gomphrena globosa* L.	Leaves	*In vivo*	Ethanol extract	Saponins, alkaloids, reducing sugars and coumarins	Oral administration to male dogs	Reduction in arterial blood pressure without change heart rate	[144]
Apiaceae	*Crithmum maritimum* L.	Leaves and stems	*In vitro*	Ethanol/water extract	Hydroxybenzoic, caffeoylquinic aand coumaroylquinic acids, esculetin, quercetin hexosides and rutin, luteolin and apigenin.	Angiotensin converting enzyme (ACE-I) inhibition	79.6% of inhibition at 0.5 mg/mL	[143]
Apocynaceae	*Apocynum venetum* L.	Leaves	*In vivo*	Water extract	n.a.	Hypertensive rat models	Reduction in blood pressure, blood urea nitrogen levels, increased urine volume, enhancement of the excretion of sodium and potassium	[145]
		Leaves	*In vivo*	n.a.	n.a.	Rat mesenteric vascular beds	Dose-dependent vasodilation mediated by endothelial cells releasing NO	[146]
		Leaves	*In vivo*	Ethanol extract	Hyperoside, isoquercitrin, procyanidins	Precontracted rat aorta	Relaxation in precontracted rat aorta and superior mesenteric artery through endothelium-derived NO	[147]
Asteraceae	*Artemisia scoparia* Waldst. & Kit.	Aerial organs	*In vivo*	Hor water extract	Eugenol 2 *O*-β-d-glucopyranoside; 4-(*O*-β-d-glucopyranoyl)-3-(3′-methyl-2′-butenyl)acetophenone; salicylic acid; 3,4-dihydroxybenzoic acid; caffeic acid; isofraxidin 7-*O*-β-d-glucopyranoside, and 4′-*O*-β-d-glucopyranoyl (*E*)-4-hydroxy-3-methylbut-2-enyl benzoate	Spontaneously hypertensive rats oral administration	Reduction in blood pressure via the enhancement of VEGF expression and the suppression of RhoA expression by reduced ACE activity and angiotensin II production	[148]
	*Chrysanthemum indicum* L.	Flowers	*In vivo*	Linarin enriched extract	Linarin	CODA Mouse & Rat Tail-Cuff Blood Pressure System	Reduction in blood pressure, inhibition of renin–angiotensin system (RAS) in kidney	[149]
Juncaginaceae	*Triglochin maritima* L.	Leaves and stems	*In vitro*	Ethanol/water (1/1) extract	Hydroxybenzoic and caffeic acids, esculetin, apigenin hexoside and luteolin	ACE-I inhibition	90% inhibition at 0.5 mg/mL	[150]
Cardiovascular disorders								
Chenopodiaceae	*Salicornia ramosissima* (Hook.f.) J.Woods ex W.A.Clarke & E.S.Marshall	Aerial parts	*In vivo*	Aqueous, hydroalcoholic, and ethanol extracts	Caffeoylquinic acid derivatives, flavonoids, and fatty acids	Transient middle cerebral artery occlusion mice model	Reduction in infarct volume and of the plasma levels of the DNA peroxidant product 8-hydroxydeoxyguanosine	[151]
Malvaceae	*Thespesia populnea* (L.) Sol. ex Corrêa	Leaves	*In vivo*	Hydroalcoholic extract	Flavonols (kaempferol)	Male adult Wistar rats submitted to doxorubicin induced citotoxicity	Reduction in heart dysfunction	[94]
Obesity								
Aizoaceae	*Tetragonia tetragonoides* (Pall.) Kuntze	Leaves	*In vivo*	Hydroalcoholic (70%) extract	6-methoxykaempferol-3-*O*-β-D-glucosyl(1‴→2″)-β-D-glucopyranoside, 6-methoxykaempferol-3-*O*-β-D-glucosyl(1‴→2″)-β-D-glucopyranosyl-(6′′′′-caffeoyl)-7-*O*-β-Dglucopyranoside, and 6,4′ -dimethoxykaempferol-3-*O*-β-D-glucosyl(1‴→2″)-β-D-glucopyranosyl- (6′′′′-caffeoyl)-7-*O*-β-D-glucopyranoside	High-fat diet (HFD)-induced obese mice	Decreased body weight gain, total white adipose tissue (WAT), liver weight, and size of adipocytes and improved plasma lipid profiles	[152]
Amaranthaceae	*Bassia scoparia* (L.) Voss (=*Kochia scoparia* (L.) Schrad.)	Fruit	*In vivo*	Ethanol extract	Total saponins, momordin Ic, 2′-*O*-β-D-glucopyranosyl momordin Ic and 2′-*O*-ββ-D-glucopyranosyl momordin IIc	Obesity model induced in female ICR strain mice and formale Wistar King mice by a high-fat diet	Inhibition of pancreatic lipase activity and reduction in plasma triacylglycerol level	[117]
	*S. europaea* (=*S. herbacea*)	Aerial organs	*In vivo*	Desalted power	Trans-ferulic acid	Obesity model induced on Sprague–Dawley rats by a high-fat diet	Reduction in obesity and adipogenesis	[78]
		Aerial parts	*In vivo*	50% ethanol extract	-	Hyperlipidemia induced by high fat diet in ICR mice	Prevention of hyperlipidemia via modulation of hepatic lipid homeostasis and regulation of SREBP-1a, FAS, GAPT, and PEPCK expression	[135]
		Aerial parts	*In vivo*	Dried powder of whole plant	-	Male ICR mice fed with high-fat diet	Decreased body weight gain, liver weight, hepatic triglyceride, serum leptin and insulin, along with the mRNA level of key lipid anabolic genes such as SREBP-1c, PPAR and FAS	[134]
Asteraceae	*P. indica*	leaves	*In vitro*	Hot water extract	3,5-*O*-dicaffeoylquinic acid	Inhibition of adipogenesis of 3T3-L1 cells and of pancreatic lipase enzyme.	Decrease in the accumulation of lipids, suppression of adipogenesis, and modified protein, carbohydrate, glycogen, and nucleic acid concentrations in the 3T3-L1 adipocytes	[140]
		Leaves	*In vivo*	Hot water extract	Phenolic and flavonoids, caffeoylquinic acid derivatives, beta-caryophyllene, and gamma-gurjunene	High-fat-diet-induced (HFD) mice	Attenuation of dyslipidemia and obesity, with reduced TG, TC, LDL-C, and perigonadal fat weight	[140]
Malvaceae	*T. populnea*	Stem bark	*In vivo*	Ethanol extract	Quercetin	Obese female albino Wistar rats pretreated with orlistat	Reduction in weight gain, adiposity level, and total cholesterol and triglyceride levels	[93]
Nitrariaceae	*Nitraria retusa* (Forssk.) Asch.	Shoots	*In vivo*	Ethanol extract	Quercetin, isorhamnetin-3-*O*-glucoside, isorhamnetin-3-*O*-rutinoside	BKS.Cg-*Dock7^m^*+/+ *Lepr*^db^/J mice model	Suppression of the increases in body and fat mass weight, decreased triglycerides and LDL-cholesterol levels, enhancement of gene expression related to lipid homeostasis in liver	[153]
		Aerial parts	*In vivo*	Infusion	Isorhamnetin	Female and male volunteers, including healthy subjects and overweight/obese participants	Obese participants experienced a significant decrease in triglyceride levels and an increase in HDL levels	[154]
		Aerial parts	*In vivo*	Infusion	Isorhamnetin	Overweight/obese women	Significant weight reduction, decrease in BMI and BF%, increase in the percentage of body water (BW), and in the ratio of LBM/BF. Significant reduction in Triglyceride (TG) levels	[155]
Plumbaginaceae	*Limonium tetragonum* (Thunb.) Bullock	Aerial parts	*In vivo*	Ethyl acetate-soluble fraction	(-)-epigallocatechin-3-(3″-*O*-methyl) gallate, (-)-epigallocatechin-3-gallate, and myricetin-3-*O*-β-D-galactopyranoside	High-fat diet-induced obese mice	Prevention of body weight gain and adipose tissue, decrease in triglyceride and total cholesterol; improvement of glucose tolerance and insulin resistance	[156]
		Aerial parts	*In vitro*	Ethyl acetate-soluble fraction	(-)-epigallocatechin-3-(3″-*O*-methyl) gallate, (-)-epigallocatechin-3-gallate, and myricetin-3-*O*-β-D-galactopyranoside	3T3-L1 adipocytes	Inhibition of adipocyte differentiation, likely mediated by the downregulation of adipogenesis-related transcription factors—PPARγ, C/EBPα, and SREBP-1—and adipocyte-specific proteins, including FAS, LPL, and aP2	[156]

n.a.: data not available; -: not applicable.

## Data Availability

No new data were created or analyzed in this study. All information discussed in this review was obtained from previously published studies cited in the article. Data sharing is not applicable to this article.
